# Antecedents of well-being of home care workers: a mixed-methods systematic review

**DOI:** 10.1093/geront/gnag131

**Published:** 2026-06-20

**Authors:** Veronika Kuradchik-Pekarskaya, Ines Martinez-Corts, Celia Gago-Valle, Francisco J Medina

**Affiliations:** Departamento de Psicología Social, Facultad de Psicología, Universidad de Sevilla, C. Camilo José Cela, s/n, 41018 Seville, Spain; Departamento de Psicología Social, Facultad de Psicología, Universidad de Sevilla, C. Camilo José Cela, s/n, 41018 Seville, Spain; Departamento de Psicología Social, Facultad de Psicología, Universidad de Sevilla, C. Camilo José Cela, s/n, 41018 Seville, Spain; Departamento de Psicología Social, Facultad de Psicología, Universidad de Sevilla, C. Camilo José Cela, s/n, 41018 Seville, Spain

**Keywords:** Carers, Vulnerable populations, Occupational health, Mental health, Quality of care

## Abstract

**Background and Objectives:**

Demand for home-based care is growing, yet the attractiveness of this sector remains low due to poor working conditions. This threatens the sustainability of this service, which is often preferred by older adults. To address this issue, it is necessary to understand of the home care environment. Thus, the aim of this review is to develop a theory-informed framework that explains how multilevel conditions influence the well-being of home care workers.

**Research Design and Methods:**

A mixed methods systematic review was conducted. The last updated search was conducted on April 8, 2026. Searches covered Jan 2000–April 2026. 2 reviewers independently coded the data, synthesized the findings using a convergent integrated approach, and integrated the evidence using the Job Demands–Resources and Work–Home Resources theories.

**Results:**

Seventy-nine studies were included. High workload volume and intensity and, emotional demands with limited organizational resources were linked to strain, while adequate supervision, information flow, training, access to material resources, and supportive relationships mitigated harm and sustained motivation. The patterns were most pronounced among direct-hire and migrant workers. Negative consequences of the devaluation of care work and the crucial role of legal rights awareness emerged as novel contributions to the field.

**Discussion and Implications:**

This theory-informed review addresses fragmented evidence on home care workers’ well-being, identifies where demand rises and resources thin under efficiency pressures, and points decision-makers—especially in direct-hire and migrant-reliant systems—to specific practices for retaining the workforce and safeguarding care quality.

## Background and objectives

The aging population and the increasing rate of chronic conditions have placed health care systems under pressure (Jane Osareme et al., 2024). Consequently, there has been a shift away from institutional care models towards home-based care (HBC), where long-term care (LTC) services are provided in the individual’s own home. Home-based care occupations span health and social care roles. The formal long-term care workforce is composed mainly of nurses and personal care workers, such as home care aides, home health aides, care assistants, and live-in carers. It also includes nurses, such as professional nurses, practical, and community nurses, as well as paid domestic workers. Broader definitions may also include social workers and rehabilitation specialists (OECD, 2020, 2023).

Home-based care is becoming the preferred choice because it upholds personal autonomy in later life and helps maintain community integration (Kueakomoldej et al., 2025; Saari et al., 2023). However, it requires a greater number of care professionals with specific skills, which is currently a challenge for many Organisation for Economic Co-operation and Development (OECD) countries and the wider issues of retention and staff turnover observed in the direct care workforce within long-term care settings (O’Keefe et al., 2025). This is related to the health-impairing working conditions in the LTC sector. Issues include low wages, limited opportunities for professional development, and lack of support (OECD, 2020). More broadly, LTC scholarship has also described direct care work as persistently undervalued despite its physical demands and considerable relational and emotional complexity (Scales, 2021). Studying and promoting both the working conditions and the well-being of care workers is critical not only to improve the quality of care provided to LTC receivers but also to safeguard the well-being of the workers themselves and to ensure the sustainability of the care systems.

Compared with those employed in institutional settings, LTC workers in HBC report worse physical and mental health (Gebhard & Herz, 2023). An explanation is the limited control that home care workers (HCWs) have over their working environment. They are exposed to safety hazards and unpredictable risks, such as second-hand smoke, aggressive pets, and unsafe conditions, often without support to manage these demands (Grønoset Grasmo et al., 2021). Home care workers also deal with psychosocial risks, such as emotionally taxing situations (e.g., death of clients), exclusion from discussions about their clients’ care plans, and a lack of specific training (Forward et al., 2024). This already precarious situation is compounded by structural inequalities that disproportionately affect women and migrants, who make up a significant proportion of the HBC workforce, resulting in worker exploitation, overwork, and sexual harassment. In addition, such issues are underreported due to the isolative nature of HBC work (Fisher, 2021). The intersecting influences of gender and ethnic origin also contribute to the undervaluing of care work and increase the risk of discrimination, often rooted in racialized and gendered perceptions of caregiving (Curington, 2020; Fisher, 2021). Among these challenges, formal protections and opportunities for collective bargaining are still limited (OECD, 2023).

Research on HCWs has expanded over the years. However, existing reviews remain fragmented and topic-specific. For instance, previous syntheses have focused on isolated challenges such as second-hand smoke exposure (Angus & Semple, 2019), end-of-life support needs (Forward et al., 2024), coping with client death (Findlay & Robertson, 2024), workplace violence (Clari et al., 2020; Phoo & Reid, 2022; Zhong & Shorey, 2023), and health interventions (Gebhard & Herz, 2023). Others, like Grønoset Grasmo et al. (2021), have explored occupational health experiences of home health aides only through the synthesis of qualitative evidence, while broader reviews on staff retention (Thwaites et al., 2023) have mainly addressed residential care settings, providing limited insight into HBC. For example, recent evidence on personal care workers’ intentions to quit working in nursing homes has highlighted the role of workplace conditions, leadership, empowerment, client–carer relationships, and societal perceptions, but offers only limited insight into the specific demands and resources of HBC (O’Keefe et al., 2025). To our knowledge, no review to date has provided an integrative, theory-informed synthesis of the full range of antecedents that relate to HCW well-being. Beyond informing policy and organizational practice, such a review is crucial for advancing research by consolidating scattered findings, identifying conceptual gaps, and setting a clearer agenda for future studies.

The use of the Job Demands–Resources theory (JD–R) (Bakker & Demerouti, 2018) and the Work–home Resources (W–HR) model (ten Brummelhuis & Bakker, 2012), both grounded in the same theoretical background, offers a much-needed framework to identify these antecedents. The JD–R theory (Bakker & Demerouti, 2018) explains how job demands or energy-consuming aspects of a job (e.g., overload) and job resources or motivating aspects (e.g., support) influence workers’ well-being. The theory distinguishes between two complementary processes: a health-impairment pathway, in which high job demands trigger strain-related mechanisms that undermine well-being, and a motivational pathway, in which job and personal resources activate motivational mechanisms that enhance well-being. In addition, employees can influence their demands and resources through proactive behaviors. Finally, demands and resources exist at multiple levels (i.e., individual, team, and organizational). While the JD–R theory is versatile, its primary focus is on factors within the work domain. A potential limitation of relying solely on a work-centric theory is the risk of overlooking well-being antecedents that exist outside the workplace, as the HBC work often entails blurred boundaries between the work and non-work domains (Grønoset Grasmo et al., 2021). For instance, research has demonstrated that emotional states can spill over from one domain to another (e.g., Martinez-Corts et al., 2015). Thus, the W–HR (ten Brummelhuis & Bakker, 2012) model is integrated to provide a comprehensive view. This model offers a resource-based perspective on the work–non-work interface. It proposes that resources and demands from one domain can influence outcomes in the other, leading to work–home conflict or work–home enrichment processes. The model categorizes these resources into three levels: macro (e.g., cultural or economic circumstances); social (e.g., support from family or supervisors); and individual (e.g., personal resources).

This review takes an integrative approach, combining these two frameworks. The JD–R theory provides the structure for integrating the job demands and resources inherent to HBC work, whereas the W–HR model allows the systematic inclusion of relevant non-work factors. This integrated approach is particularly relevant in the HBC sector, where organizational support is limited. As a result, more isolated HCWs, such as live-in and migrant workers, often rely on personal and community-based social networks as a valuable resource (van Bochove & zur Kleinsmiede, 2021). Therefore, including this dual-theory framework provides a robust structure for categorizing emergent themes from the evidence and understanding how they relate to well-being.

### Objectives

This systematic review aims to outline a conceptual framework that explains how personal and contextual factors influence the well-being of HCWs. To this end, primary evidence from quantitative, qualitative, and mixed-methods studies will be synthesized using the categories of the JD–R theory and the W–HR model to identify the multilevel factors contributing to the HCW well-being outcomes. The review will also identify sources of vulnerability in the sector, considering how differences in certain working conditions (e.g., agency vs. direct employment) may influence workers’ experiences.

## Research design and methods

### Design

This review followed the Joanna Briggs Institute guidelines for mixed methods systematic reviews (MMSR) using a convergent integrated approach for thematic analysis (Lizarondo et al., 2024). The results were categorized according to the JD–R and W–HR theoretical frameworks. The protocol was registered on PROSPERO (CRD42023492438) and followed the PRISMA protocol (Page et al., 2021) (see Supplementary Material, File 1).

### Search methods

A preliminary scan of the literature informed the keywords selection. An initial limited search was performed to identify relevant keywords in titles and abstracts, as well as index terms. In addition, occupational terminology used in relevant papers was reviewed, and database indexing terms, including medical subject headings in MEDLINE, were consulted to identify standardized descriptors related to paid HBC work. These terms were organized into three groups: (a) keywords identifying the target of this study (HCWs), (b) well-being outcomes, and (c) antecedents. The SPIDER tool (Cooke et al., 2012) was used to guide the search strategy and retrieve primary evidence studies (see Table 1). The strategy was adapted to each database. A university librarian and two coauthors with expertise in evidence synthesis and database searching reviewed the final search strategy. The complete search strategies for all databases can be found in Supplementary Material, File 2.

**Table 1:** Search strategy guided by the SPIDER tool.

SPIDER* element	Criteria	Search strategy
**Sample**	Studies targeting paid long-term care workers employed in home-based care settings. The article should provide disaggregated results for home care workers if they include more occupational groups in its sample	Abstract(“home caregiver*” OR “domestic service worker*” OR “professional carer*” OR “homebound care” OR “health care service*” OR “basic care worker*” OR “home care” OR “paid caregiver*” OR “remunerated caregiver*” OR “professional caregiver*” OR “formal caregiver*” OR “personal care aide*” OR “professional home care provider*” OR “domestic care worker*” OR “direct care worker*” OR “paid home care staff” OR “home health aide*” OR “home support worker*” OR “home health care” OR “home nurs*” OR “home health nurs*” OR “visiting nurs*” OR “community nurs*”)
**Phenomenon of interest**	Antecedents of well-being (e.g., working conditions, demands and resources)	AND Abstract(“working conditions” OR “emotional adjustment” OR “emotional regulation” OR “self-care” OR “self-efficacy” OR “empowerment” OR “self-management” OR “coping behavior” OR “psychosocial factors” OR “motivation”)
**Design**	Any empirical study using interviews, focus groups, surveys, case studies, etc.	All empirical study designs were considered for inclusion
**Evaluation**	Outcomes related to physical, and mental or emotional well-being	AND Abstract(“well-being” OR “health” OR “physical health” OR “mental health” OR “stress” OR “burnout” OR “anxiety” OR “depression” OR “satisfaction” OR “life satisfaction” OR “quality of life”)
**Research**	Primary evidence using qualitative, quantitative, or mixed methods published in academic journals. Literature reviews and intervention studies were excluded	AND (stype.exact(“Scholarly Journals”) Where the functionality was available, a filter was used to specifically exclude publication types such as literature reviews, systematic reviews, and meta-analyses
**Other criteria**	Articles in English or Spanish, and published between 2000 and the present day (July 2025)	AND la.exact(“ENG” OR “SPA”) AND yr(2000–2029))

*Note*. *SPIDER = Sample, Phenomenon of Interest, Design, Evaluation, and Research type.

The last updated search was conducted on April 8, 2026. Reference screening and citation tracking (from Grønoset Grasmo et al., 2021 and other manually searched articles) were also performed. Searches were conducted in Clarivate Web of Science, ProQuest PsycINFO, Ovid MEDLINE, and EBSCOhost CINAHL Complete, covering publications from January 2000 to April 2026. The year 2000 was chosen as the cut-off point to ensure the inclusion of research conducted within the context of modern LTC systems and labor policies, considering the expansion of marketized care, migrant care labor, and the development of HBC services over the last two decades.

### Inclusion and exclusion criteria

This review focused on paid HCWs providing HBC to older adults or other adults with ongoing care needs. This included agency-employed HCWs and those hired directly. As primary studies did not consistently report care duration or formal service classification, eligibility was determined based on the context of the study, population receiving care, and nature of the work described. Thus, studies were included in the review if they were empirical (i.e., quantitative, qualitative, or mixed methods), focused on paid HCWs (e.g., nursing staff and personal assistants), and examined potential antecedents of well-being. These antecedents could be psychosocial or organizational factors, job demands and resources, or individual characteristics. The studies had to be published in English or Spanish.

Studies focusing on unpaid domestic labor, institutional settings or, outcomes unrelated to well-being were excluded. Pediatric HBC was excluded from the scope of this review, as it involves distinct care dynamics, clinical demands, and family interactions that differ substantially from adult care and would introduce heterogeneity not aligned with the review’s focus. So were the literature reviews, books, and other nonempirical publications. Reviews were excluded to prevent duplication of primary data, as the aim of this review was to extract, appraise, and synthesize empirical original study evidence. As the review focused on identifying and synthesizing antecedents of HCW well-being rather than evaluating intervention effects, intervention studies were excluded. Articles were excluded if HCWs were included alongside other occupational groups, and disaggregated findings specific to them were not reported.

### Search outcome

One reviewer screened the titles, abstracts, and full text of all records identified against the eligibility criteria after duplicate removal (*n* = 2,386). To ensure the reliability of the screening process, a second reviewer independently screened 43.5% of the total records (*n* = 1,039 out of 2,386) as full dual screening was not feasible. While there is no standardized percentage for partial dual review, the Cochrane guide for rapid reviews recommends a minimum of 20% (Garritty et al., 2021). Disagreements were resolved through discussion or by involving a third reviewer.

### Quality appraisal

The quality of the included studies was assessed using the Mixed Methods Appraisal Tool (MMAT) (Hong et al., 2018). Each study was appraised by two independent reviewers. Disagreements were resolved through discussion.

### Data abstraction

The data were extracted using a structured form covering the following: author, year, country, design, aim, theoretical framework, employment characteristics of participants, migration status, and main findings relevant to the review question. The extracted qualitative data findings consisted of themes or subthemes reported by the authors, together with supporting quotations from participants, observations, or other illustrations where available. For quantitative data, the extracted findings included all results relevant to the review question. These results included descriptive data and inferential results and reported both significant and nonsignificant associations between work-related factors and workers’ well-being. All studies were independently coded by two reviewers in ATLAS.ti 9. Disagreements were resolved through discussion, and unresolved decisions were reviewed by a third author.

### Synthesis

In line with a convergent integrated approach, the quantitative findings were transformed into “qualitized” narrative statements to facilitate integration with the qualitative data. This transformation involved turning quantitative results into concise textual statements that kept the direction, importance, and context of the original findings while avoiding reinterpreting them as cause and effect beyond the scope of the primary studies. For example, statistically significant associations were expressed as statements such as “[relevant factor] was (or was not) associated with [well-being outcome],” while descriptive results were transformed into narrative summaries such as “[specific demand or resource] was commonly reported among workers.” After this “qualitization” of quantitative data, qualitative and quantitative findings were assembled into a single textual data set for analysis.

The initial deductive dimensions were informed by the JD–R and W–HR theories, while the themes and codes were developed and refined through repeated inductive comparisons of the qualitative and “qualitized” quantitative findings. Codes were grouped into categories based on similarity of meaning, and these categories were then synthesized into higher-order, integrated themes. The two reviewers agreed on the decisions regarding integration, which were then presented and discussed with the other contributors. When a finding could fit multiple codes, its placement was decided based on its primary meaning and relevance. Disagreements were reviewed by a third author.

## Results

### Study selection

**Figure 1:**
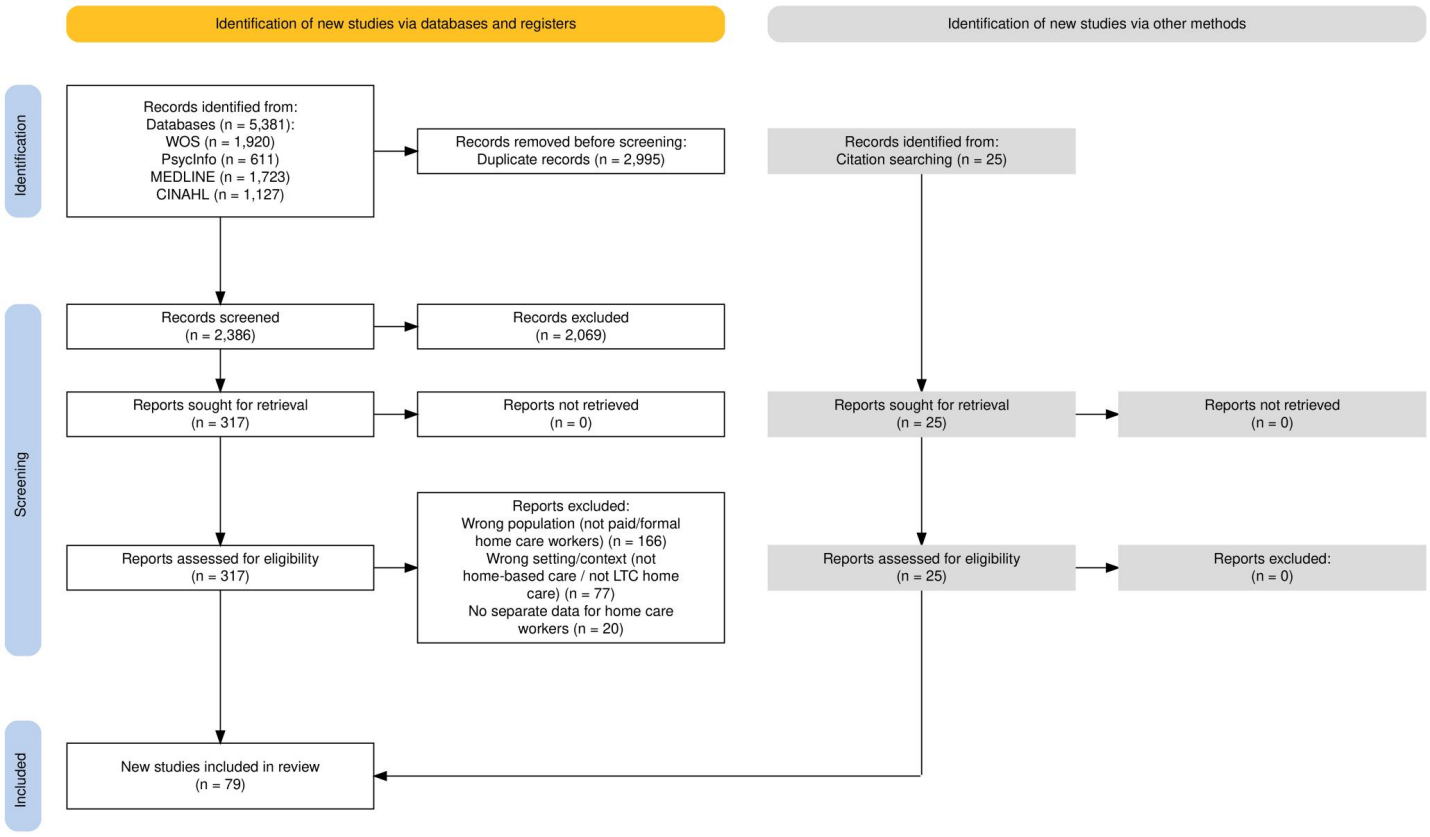
PRISMA flow diagram note: The diagram was elaborated using PRISMA’s shiny app (Haddaway et al., 2022).

A total of 5,381 records were identified through database searches (see Figure 1). An additional 25 records were identified through citation screening. After removing duplicates, 2,386 records were screened, resulting in the exclusion of 2,069. A total of 317 reports from database searches and 25 from citation screening were retrieved and assessed for eligibility. At this stage, 263 studies were excluded for the following reasons: wrong target population (*n* = 166); wrong employment setting (*n* = 77); and the study did not disaggregate findings for HCWs (*n* = 20). Following critical appraisal, 79 studies were included in the review: 54 from database searches and 25 from citation screening.

### Study characteristics

Study-level characteristics are in Supplementary Material, File 3. The sample included 32 quantitative, 31 qualitative, and 16 mixed-methods studies (see Table 2). Among the studies with quantitative data collection, 85.42% used cross-sectional designs. Only a few used longitudinal designs (n = 7). Almost half of the studies were conducted in North America, followed by Northern and Western Europe.

**Table 2:** Study designs, locations, and workforce characteristics in the included studies.

Variable	Category	Subcategory	Frequency	Percentage
**Type of study**	Quantitative		32	40.51
	Qualitative		31	39.24
	Mixed-method		16	20,25
	Total		79	100
Method of quantitative data collection*	Cross-sectional		41	85.42
	Longitudinal		7	14.58
	Total		48	100
**Geographic location**	North America	United States and Canada	34	43.04
	Northern Europe	Sweden, Norway, Finland, and Denmark	15	18.99
	Western Europe	United Kingdom, France, Germany, and Netherlands	10	12.66
	Southern Europe	Spain, Italy, and Croatia	9	11.39
	EU*	Belgium, Germany, France, Italy, Netherlands, Poland, and Slovakia	1	1.27
	East Asia	China, South Korea, and Taiwan	5	6.33
	Middle East	Israel	3	3.8
	Oceania	Australia and Aotearoa (New Zealand)	2	2.53
	Total		79	100
**Theoretical framework**	Theory-informed study		44	55.7
	Non-theoretical study		35	44.3
	Total		69	100
Studies’ participants’ employment type*	Mixed employment arrangements		21	26.58
	Public-sector employment		19	24.05
	Agency-based employment (unspecified)		25	31.65
	Direct employment by client or family		9	11.39
	Private-sector employment		3	3.8
	Worker-owned cooperative		1	1.27
	Unspecified employment arrangement		1	1.27
	Total		79	100
Studies’ participants’ HBC arrangement*	Live-out		50	63.29
	Mixed HBC arrangements		10	12.66
	Unclear		13	16.46
	Live-in		6	7.59
	Total		79	100
Studies’ participants’ HBC occupation*	Personal care workers		56	70.89
	Mixed occupational groups		15	18.99
	Nursing health professionals		8	10.13
	Total		79	100
Studies’ participants’ migration status*	Not specified		44	55.7
	Mixed sample		24	30.38
	Migrant HCWs		10	12.66
	Nonmigrant HCWs		1	1.27
	Total		79	100

*Note*. *Categories marked with an asterisk are further defined in this note. HBC = home-based care; HCW = home care worker. Method of quantitative data collection refers to the quantitative data from quantitative and mixed-method studies. EU = is a specific study that was conducted in numerous EU countries, including Western, Southern, and Eastern Europe. Studies’ participants’ employment type: *Public-sector employment—*employed directly by a government or public authority; *Private-sector employment—*employed by for-profit companies or organizations operating in the private market; *Agency-based employment—*not specified if public or private—employed through agencies, but the agency’s status as public, private, or non-profit is not clearly specified in the study; *Worker-owned cooperative—*workers are part of a cooperative model where they share ownership and decision-making power; *Direct employment by client or family—*hired directly by the client or their family, without any agency; *Mixed employment arrangements—*employed under more than one model (e.g., some work for agencies, others are directly hired); *Unspecified employment arrangement—*insufficient information to determine the employment model or relationship. Studies’ participants’ HBC arrangement: *Live-in—*the HCW lives in the same household as the care recipient, often providing round-the-clock care or residing with the client during the employment period; Live-out—the HCW visits the client’s home to provide care but lives elsewhere; care is typically provided in shifts or scheduled visits; Mixed *arrangements—*the study includes both live-in and live-out arrangements, or explicitly mentions both types among participants; *Unspecified—*The study does not provide sufficient detail to determine whether workers live with clients or not. Studies’ participants’ HBC occupation: *Personal care workers—*non-clinical workers who provide support with daily activities such as bathing, dressing, feeding, cleaning, and companionship in HBC settings, and they typically hold no formal health care license and are often referred to as aides or assistants; *Nursing health professionals—*licensed professionals trained to provide clinical or nursing care, including tasks such as health assessments, medication administration, wound care, and health education, and they usually hold nursing or allied health qualifications; *Mixed occupational groups—*samples that include both personal care workers and clinical professionals, or broader teams encompassing multiple roles in home care (e.g., aides, nurses, therapists, coordinators, etc.). Studies’ participants’ migration status: *Migrant HCWs*—All participants are foreign-born or identified as international migrants, often with specific migration backgrounds; *Nonmigrant HCWs—*all participants are native-born workers in the country where the study was conducted; *Mixed sample—*the sample includes both migrant and nonmigrant workers, or migration status is reported for a portion of participants; *Unspecified—*the study does not provide information about participants’ migration background or country of origin.

Most participants were hired through agencies in either public, private, or unspecified agency settings. Of this, 26.58% involved mixed employment models, combining agency and direct-hire working arrangements. Fewer studies examined direct employment by clients (*n* = 9) or worker-owned cooperatives (*n* = 1). The majority focused on personal care workers in live-out HBC arrangements. Migration status was not reported in over half of the studies. When stated, workers were typically part of mixed migrant/nonmigrant samples. Of the studies included, 44.30% were not theory-informed. The most employed theories were stress models (e.g., JD–R and Job Demand–Control–Support), whereas only six used frameworks related to structural inequality, gender, or feminist theory (see Table 3).

**Table 3:** Theories and conceptual frameworks employed by theory-informed studies (*N* = 37).

Theoretical group	Definition	Specific theories/models used	Frequency*	Studies N°*
**Occupational stress models**	Frameworks focusing on the relationship between job demands, control, effort, reward, and social support to explain occupational strain and its health impacts	Job demand–control model, job demands–resources model (JD–R), allostatic load model, compensatory control theory, effort–reward imbalance model	17	3, 7, 16, 17, 18, 37, 38, 39, 57, 60, 61, 63, 64, 66, 68, 75, 79
**Health psychology and biopsychosocial approaches**	Psychological and behavioral models that address individual responses to stress, coping, health behavior, and the interaction between physical and mental health	Self-efficacy theory, social cognitive theory, stress and coping theory, biopsychosocial model, safety climate theory, psychological resilience, agency, risk information seeking and processing model	9	26, 33, 34, 37, 40, 70, 75, 76, 77
**Sociological and interactionist theories**	Grounded theory and symbolic interactionism approaches that inductively explore social meaning and interaction in care work	Grounded theory, Cohen’s phenomenological methodology, constructivist research paradigm, collective sensemaking	7	9, 13, 22, 30, 51, 69, 73
**Inequality, feminist, and gender theories**	Theories examining how gender, power, and structural inequalities shape labor conditions, care work, and workers’ vulnerability	Multiple disadvantage frameworks, feminist political economy, emotional labor, intersectional assemblage theory, flexibility–security matrix, transnational social inequality	6	8, 22, 24, 32, 48, 75
**Organizational behavior and empowerment theories**	Approaches that focus on empowerment, control, motivation, team climate, and organizational justice within the workplace	Empowerment theory, organizational justice model, attribution theory, action regulation theory, job characteristics model, employee engagement theory, meaning of work	6	2, 14, 35, 53, 60, 63
**Ecological and multilevel frameworks**	Theories analysing how health and well-being are shaped by individual, organizational, and systemic-level factors simultaneously	Social ecological model, multilevel stressor frameworks, unified concept of worker well-being	4	5, 22, 56, 71
**Other/study-specific conceptual models**	Conceptual models developed or adapted for specific studies, combining elements of existing frameworks or proposing unique lenses	Modified reasoned action approach, conceptual model of direct care worker job outcomes, organizational discrepancy	3	2, 25, 36

*Note*. Frequencies indicate how many of the theory-informed studies employed each theoretical group. As some studies used more than one theoretical framework, the total number of occurrences exceeds the number of theory-informed studies included in the review. “Study Nº” refers to the identifiers assigned to the 79 included articles, which can be matched to each article using the first column (“Nº”) of Supplementary Material, File 3.

### Risk of bias

No studies were excluded based on methodological quality. All 31 qualitative studies met the appraisal criteria. The 31 quantitative studies (24 non-randomized and 8 descriptive) were generally of moderate to high quality. The most common weakness was insufficient reporting, particularly regarding sample representativeness and the risk of response bias. Most of the 16 mixed-methods studies adequately justified their design and integrated the qualitative and quantitative findings. Only a few showed limitations in the quantitative part of their studies that affected their quality scores. Overall, the quality of the studies included in the review was moderate to high. Full MMAT results are presented in the Supplementary Materials (Supplementary File 4).

### Results of syntheses

**Figure 2:**
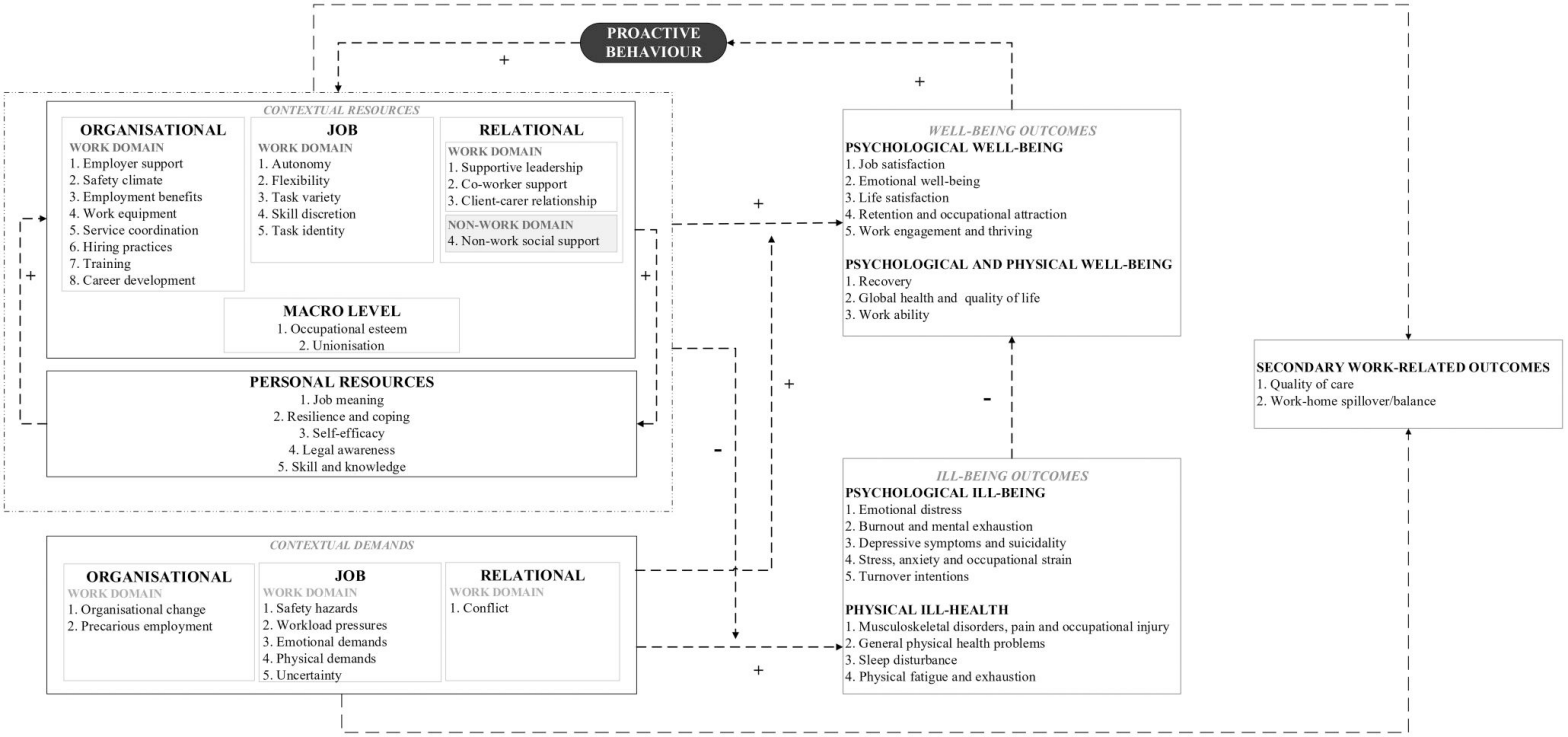
The conceptual framework of the factors influencing the well-being of home care workers.

Figure 2 presents the conceptual model with the identified themes (see also Table 4 for definitions). Well-being outcomes were grouped into both positive (i.e., well-being outcomes) and negative (i.e., ill-being outcomes) indicators. Contextual factors were grouped into four domains: job-related (i.e., job characteristics); organizational (i.e., factors related to the employer, who can be either an agency or an individual provider, such as the HBC receiver); relational (e.g., workplace and non-work relationships); and macro factors (e.g., sociocultural aspects). Worker-related factors were categorized as personal resources and proactive behaviors.

**Table 4:** Themes and codes identified through synthesis.

Dimension	Theme	Code	Code definition	Study N°*
**Ill-being outcomes**	Psychological and occupational ill-being. A set of negative emotional experiences, stress responses, and mental health symptoms connected to home care work, indicating poor occupationalpsychological well-being	Emotional distress	Negative emotional experiences that are not necessarily clinical in nature, such as frustration, sadness, guilt, homesickness, worry, or general emotional distress arising from home care work	2, 3, 9, 13, 15, 17, 22, 29, 33, 34, 40, 42, 47, 53, 57, 71, 74, 75
		Burnout and mental exhaustion	Work-related occupational burnout syndrome, characterized by emotional or mental exhaustion and, when assessed, also by depersonalization/cynicism and reduced professional fulfilment	4, 7, 10, 11, 12, 26, 30, 41, 49, 62, 71, 72, 79
		Depressive symptoms and suicidality	Depressive symptoms and signs of suicidal tendencies, including low mood, anhedonia, and suicidal ideation	5, 17, 26, 58
		Stress, anxiety, and occupational strain	Work-related reactions of arousal, tension, anxiety, and psychological overload, including perceived stress, psychological distress, work-related fear (e.g., of hurting or being hurt by clients) and occupational strain	9, 11, 12, 18, 20, 21, 22, 26, 37, 39, 42, 44, 50, 52, 54, 63, 64, 66, 71, 73, 74, 75, 79
		Turnover intentions	Thoughts, intentions, or plans to leave one’s current job or profession, understood as negative indicators of occupational well-being and job disengagement	6, 10, 11, 12, 15, 21, 22, 31, 34, 35, 38, 39, 46, 60, 61, 64, 76, 78, 79
	Physical ill-health. A set of physical symptoms associated with home care work, including fatigue, illness, pain, and injuries.	Musculoskeletal disorders, pain, and occupational injury	Musculoskeletal problems and work-related physical injuries, including pain, disorders, functional disability and acute occupational injuries	1, 3, 4, 10, 12, 17, 18, 30, 31, 36, 37, 38, 39, 41, 44, 46, 49, 52, 58, 59, 65, 66, 79
		General and non-musculoskeletal physical health problems	Work-related physical ill-health outcomes involving non-musculoskeletal conditions or symptoms, such as respiratory, cardiovascular, dermatological, or urological problems; psychosomatic symptoms; poorer self-rated general health; and consequences related to illness, such as sick leave and sickness presenteeism	1, 2, 3, 10, 17, 18, 23, 27, 30, 35, 47, 56, 58, 60, 77
		Sleep disturbance	Work-related disturbances in sleep quality, sleep maintenance, or the ability to fall asleep	3, 15, 26, 41
		Physical fatigue and exhaustion	Physical exertion, bodily fatigue, or tiredness resulting from the occupational physical activity	40, 49, 50, 54, 69, 72
**Well-being outcomes**	Psychological and physical well-being. Broad indicators of well-being, recovery and functioning which consider overall physical and psychological health, as well as perceived ability to continue working.	Recovery	The ability to rest and replenish physical and psychological resources following physical exertion and work-related stress	3, 17, 69
		Global health and health-related quality of life	Overall perception of health status and health-related quality of life, considering physical, mental, and social factors	11, 12, 20, 68
		Work ability	Perception of one’s own ability to continue performing one’s job, considering one’s health and the balance between personal resources and work demands	36, 37
	Psychological and occupational well-being. Positive work-related attitudes and broader life evaluations that reflect satisfaction, positive emotional well-being, commitment to home care work and effective performance.	Job satisfaction	A generally positive assessment of the job or various aspects of employment, such as working conditions, working hours, support, pay, or workplace relationships.	6, 9, 10, 11, 12, 15, 16, 17, 18, 21, 28, 31, 34, 35, 46, 60, 61, 62, 63, 64, 70, 71, 75, 79
		Emotional well-being	A predominant experience of positive emotions, subjective psychological well-being or a favourable emotional state in relation to work or everyday life	34, 37, 50
		Life satisfaction	An overall positive assessment of one’s life as a whole	15, 58
		Retention and occupational attraction	Positive indicators of retention or attraction to the job or sector, including reasons for staying, intention to remain and perceived job appeal	25, 32, 74
		Work engagement and thriving	A positive state of active engagement with home care work, characterized by vigor or vitality, dedication, absorption, energy and a sense of learning or growth	14, 53
**Work-related outcomes**	Secondary work-related outcomes. Relevant work-related outcomes associated with home care workers’ well-being but not directly constituting well-being states themselves	Quality of care	Results relating to the quality, continuity, and appropriateness of the care provided to the care recipient	2, 6, 8, 9, 15, 19, 21, 35, 44, 46, 53, 63, 74, 75, 78, 79
		Work–home spillover and work–life balance	Interference, blurring of boundaries, or difficulty in distinguishing between work and personal/family life, including work–family conflict and difficulty in disconnecting after work hours	10, 11, 21, 22, 29, 34, 49, 50, 73, 74
**Macro domain**	Occupational esteem. The social value and recognition attributed to HCWs’ roles. Among personal care workers, low occupational esteem was reflected in perceptions of their work as low-skilled, leading to stigma, exclusion, and, in some cases, legal, and social vulnerability, particularly for live-in migrant workers. Outcomes tied to the theme: attraction to the sector, emotional ill-being	Perceived low social status within the health care hierarchy	HCWs, particularly personal care workers, were often viewed as occupying the lowest tier within care teams, receiving less respect than nurses or physicians despite performing complex tasks	35, 46, 71, 79
		Stigma from broader society	Being stereotyped as low-skilled, domestic laborers rather than recognized care professionals. This stigma extended to interactions with clients, families, and the public	21, 22, 31, 49, 65, 79
		Undervaluation despite increasing responsibility and skill	Some studies highlighted a growing gap between the complexity of care tasks and the lack of recognition or status accompanying them	8, 65
		Structural consequences of low esteem	Occupational devaluation had practical consequences, including difficulty attracting new workers and increased legal and social vulnerability for some groups	4, 63
	Unionization. The presence and role of unions in representing and supporting HCWs. This was associated with improved job satisfaction and employment conditions, as well as workers’ ability to advocate for their rights. This was particularly significant among personal care workers and those employed in client-hired arrangements. Outcomes tied to the theme: job satisfaction, intention to remain in the job	Instrumental and emotional support	Unions provided practical resources (e.g. training, food banks), advocacy in employment disputes, and a sense of belonging and dignity through collective identity.	17
		Influence on job satisfaction and retention	Union membership led to job satisfaction and the intention to stay in the job	17, 74
		Organizational support role under client-hired models	Even in models where workers are employed directly by clients, unions could play a supportive organizational role	31
		Risks in non-unionized or unregulated settings	In the absence of collective bargaining, workers faced unresolved contract violations and lacked mechanisms to pressure employers	48
**Organizational resources**	Employer support. Employer practices that promote feelings of value and protection among HCWs, such as emotional support, responsiveness, and task clarification. These practices have been observed across various employment arrangements, with some emerging more commonly in agency-based models. Outcomes tied to the theme: job satisfaction, turnover intentions/intentions to remain employed, emotional well-being	Perception of employer support	Impressions of employer supportiveness, beyond specific events or interactions; broad experiences of feeling cared for or supported in the role	14, 28, 38, 49, 55
		Emotional availability and listening	Employers foster emotional well-being by creating space for workers to express concerns, vent frustrations, and feel heard without judgment	29, 35
		Practical accommodations	Employers respond to individual needs by adjusting schedules, workloads, or job demands when possible	12, 18
		Support during crises or conflict	Employers intervene or offer protection during difficult client relationships or false accusations. Lack of such support contributes to burnout or leaving the job	11, 12
		Responsiveness and follow-up	Employers take workers’ concerns seriously, especially regarding disrespect, health and safety, or workplace issues	10, 30, 58, 59
		Managing client expectations	Employers support workers by helping clients understand the scope and limits of care tasks, reducing the risk of overreach and relational conflict	31, 46, 50, 67, 71, 74
	Safety climate. HCWs’ perceptions of how health and safety are prioritized and managed in their work environment Outcomes tied to the theme: work ability	Perceived safety climate and its effects	The general perception of how safe the work environment is, and how that perception relates to HCWs’ engagement in risk management activities, confidence, and ability to perform their job	36, 37
		Safety is employer’s responsibility	Belief that employers are responsible for ensuring safe working conditions and managing health and safety risks	10
		Inequities in safety climate by employment model	Differences in perceived safety climate depending on employment type, particularly between agency-hired and client-hired workers	59
	Benefits. Financial and non-financial rewards provided by employers, including employment benefits, incentives and health insurance. These were discussed across agency-based, client-hired, and mixed employment arrangements Outcomes tied to the theme: job satisfaction, turnover/retention, emotional and physical well-being	Perceived adequacy or lack of employment benefits	HCWs’ experiences of having or lacking standard employment benefits, such as paid sick leave, vacation, retirement, transport reimbursement, and other job-related rewards	8, 11, 12, 18, 21, 24, 29, 31, 46, 52, 58, 74, 78, 79
		Incentives and job-related rewards	Rewards or forms of recognition, including performance-based incentives, bonuses, or supervisor recognition, which were experienced as forms of support	29, 35
		Access to healthcare through employment	Access to health insurance and its effects on HCWs’ emotional and physical well-being, job satisfaction, and retention, especially in systems where insurance is tied to employment	11, 12, 17, 35, 46, 62, 79
	Work equipment. The availability, adequacy and appropriateness of physical tools, equipment, and supplies needed by HCWs to perform their tasks safely and effectively, including assistive devices, medical tools, and work-related materials. Outcomes tied to the theme: musculoskeletal health, emotional well-being, turnover	Availability and use of assistive devices for transfer and mobility tasks	Access to mechanical lifts, ergonomic tools, and low-tech assistive devices (e.g., grab bars, shower chairs) to safely support client mobility and prevent musculoskeletal disorders (MSDs)	9, 30, 37, 44, 46, 47, 59, 65, 66, 67, 75, 77
		Provision of materials and supplies for safe and effective care	Access to the tools and materials required to perform everyday care tasks, including protective gear, medical instruments, and disposables	3, 31, 35, 79
		Use of personal equipment and financial burden	Situations where HCWs had to use or purchase their own work-related materials (e.g. phones, data plans, transportation), often resulting in financial strain	74, 78
	Service coordination. Effectiveness of planning, scheduling, information flow, and communication among the professionals involved in agency-based HBC delivery. Outcomes tied to the theme: emotional well-being, work–home spillover, continuity and quality of care	Planning and scheduling practices	How work is organized, including time allocation, workload distribution, and whether HCWs’ input is considered	12, 50, 63
		Communication within the agency	Quality of communication between HCWs and their supervisors or management, especially regarding care responsibilities and changes	11, 16, 18, 30, 35, 46, 71, 79
		Coordination between care providers	The extent to which HCWs are included in care-related conversations and aligned with other professionals (e.g., hospitals, primary care, specialists)	9, 21, 37, 71, 74
		Emotional strain from poor management	Emotional burden experienced by HCWs in response to inefficient management. Tolerance for such issues was limited and seen as unacceptable sources of stress	75
	Hiring practices. Selection, recruitment, and onboarding management practices Outcomes tied to the theme: emotional ill-being (frustration, burnout), turnover intentions	Inadequate recruitment and onboarding	Concerns raised by agency-hired HCWs about how new personnel are selected, prepared, and introduced into the workplace. Poor recruitment and communication about job expectations were seen as contributing to coworker underperformance, burnout, and turnover	79
	Training. The provision, quality, and relevance of training and education for HCWs, including formal education, on-the-job training, and ongoing skill development Outcomes tied to the theme: emotional well-being, job satisfaction, retention/turnover, performance	Access to and quality of training	HCWs’ access to training opportunities and their perceptions of whether training is specific, practical, relevant, and sufficient to meet care demands	8, 9, 10, 15, 16, 19, 29, 30, 33, 34, 35, 37, 44, 61, 65, 71, 73, 74, 77, 78, 79
		Formal education and skill alignment	The level and relevance of formal education and vocational training for HBC work, and how well workers’ education aligns with their assigned tasks	8, 19, 21, 34, 50, 59, 62, 63, 72
		Onboarding and orientation of new hires	How new HCWs are introduced to their roles, trained by peers or supervisors, and supported during their transition into the job	9, 35, 49, 73, 74, 79
	Career development. Perceptions of career development, advancement opportunities, and professional growth within the diverse HBC settings Outcomes tied to the theme: lower cynicism and higher professional efficacy (burnout dimensions)	Limited opportunities for advancement	The extent to which HCWs feel they have access to professional growth, internal promotion, or educational advancement, and how these limitations affect well-being	7, 15, 35, 59, 78
**Organizational demands**	Organizational change. Agency-level reforms, restructuring, and cost-saving strategies that reshaped HBC delivery. These changes, often introduced without sufficient support, altered job demands, contributed to staff shortages, and deprioritized relational care in favor of efficiency Outcomes tied to the theme: emotional ill-being, sick leave, reduced quality of care	Efficiency reforms and organizational restructuring	System-level reforms, restructuring, and cost-cutting measures implemented to increase efficiency. Often introduced without adequate support, these changes led to higher workloads, stress, time pressure, and reduced focus on relational care	2, 53
		Devaluation of relational care	A perceived shift in care role values, where measurable, instrumental tasks were prioritized over emotional or relational aspects of the work. Workers felt this conflicted with the essence of care work and caused emotional strain	2, 18, 75
		Staffing shortages and workload	Chronic staff shortages and increased client loads, often intensified by organizational change, led to psychological strain and service quality concerns	9, 37, 72
	Precarious employment. The strain linked to the nature of employment contracts, irregular scheduling, insufficient protections, and low or unstable income. These conditions vary across employment types and intersect with migrant status and live-in arrangements to shape workers’ vulnerabilities Outcomes tied to the theme: stress, emotional ill-being, burnout, reduced quality of life, turnover intentions, job dissatisfaction, work–home spillover	Financial strain due to low and unstable income	Low pay and inconsistent earnings contributed to difficulties covering basic needs, or supporting family, particularly for HCWs from low-income households or migrant backgrounds	2, 8, 10, 11, 12, 15, 17, 18, 20, 21, 25, 29, 31, 34, 35, 46, 56, 69, 71, 79
		Irregular and unpredictable hours	Lack of consistency in scheduling, last-minute cancellations, or underemployment that affects HCWs’ financial security, stress levels, and personal lives	7, 11, 12, 17, 18, 19, 21, 22, 37, 49, 58, 59, 67, 69, 74, 79
		Precarious or exploitative employment terms	Insecure or strenuous job arrangements, including lack of formal contracts, unpaid overtime, part-time or temporary work, or legal ambiguities. These conditions often lead to poor protections, job insecurity, and are more acute for migrant and live-in HCWs	13, 24, 25, 32, 34, 48, 56, 58, 59, 74
**Job resources**	Autonomy. HCWs’ perceptions of control over how they carry out their tasks, including independent decision-making, task prioritization, and use of professional judgment. Outcomes tied to the theme: job satisfaction, burnout (professional efficacy and cynicism), retention, emotional ill-being, safety	Perceived autonomy and its effects	The extent to which HCWs feel they can act independently in their work, such as choosing how to perform tasks, prioritize needs, and use judgment. Autonomy was often tied to meaningfulness, job satisfaction, and emotional well-being	7, 12, 16, 18, 31, 35, 37, 46, 63, 68
		Variability in autonomy by role, type of employment or identity	Reported differences in perceived autonomy based on individual characteristics (e.g., gender, race/ethnicity—lower for women, and Hispanic), occupational group (e.g., nurses vs. aides—higher for nurses), or employment type (e.g., cooperatives, agency-hired, direct-hired—higher for cooperatives). These influenced workers’ experiences of control, retention, and motivation	10, 17, 25, 55
		Limitations in autonomy	Reports of restricted autonomy, either in the form of being confined to narrow tasks or being hindered by contextual constraints such as working in clients’ homes, having no control over planning and feeling “mastered” by clients	3, 9, 12, 17, 18, 69, 73, 74
		Definitions and domains of autonomy	How autonomy was conceptualized by studies and participants; commonly as decision authority, discretion, self-determination, or control over scheduling, methods, pace, or task focus	7, 53, 55, 64, 72
	Flexibility. HCWs’ ability to influence or adapt their work schedules in response to personal needs, client demands, or organizational structures. Flexibility was experienced both as a supportive condition (when granted) and as a job requirement (when workers had to adapt to clients’ needs) Outcomes tied to the theme: intentions to remain employed, emotional ill-being	Perceived scheduling flexibility	Flexibility perceived by HCWs as the ability to choose or influence their working hours, including when and how they work	12, 15, 31, 45, 46, 50, 59, 74
		Organizational support for flexible scheduling	Employer-driven practices that provide or respect HCWs’ flexibility preferences (e.g., shift choice, non-justified leave, schedule negotiation)	12, 50
		Flexibility as a job requirement	Flexibility demanded of HCWs due to the unpredictable nature of care work or changing client needs	19, 67
		Autonomy–flexibility interaction	Flexibility linked to broader control over one’s work and scheduling, which could enhance autonomy but also blur boundaries, increase workload, or reduce opportunities for rest	8, 46, 56
	Task variety. HCWs’ perceptions of diversity or repetitiveness in their daily tasks, client interactions, and care contexts Outcomes tied to the theme: job satisfaction, stress, intention to remain employed	Positive task variety	Work perceived as diverse or stimulating, including exposure to different clients or tasks, which supports motivation or ITR	9, 74
		Negative task repetitiveness	Work perceived as monotonous or lacking variation, which negatively affects job satisfaction and increases stress	18
	Skill discretion. The extent to which HCWs’ jobs require the use of complex skills, professional knowledge, and opportunities to learn. Skill discretion was framed either as a job requirement or a potential job resource linked to lower burnout Outcomes tied to the theme: professional efficacy and cynicism (burnout dimensions)	Skill discretion as a job requirement	The perceived need for HCWs to apply a high level of skill, judgment, or health knowledge in their everyday tasks.	8, 37
		Skill discretion as a job resource	Skill discretion conceptualized as a job resource that may reduce emotional exhaustion and improve motivation and performance	7
		Skill discretion with no observed outcomes	Studies that examined skill discretion found no significant associations with well-being or organizational outcomes (job satisfaction, stress, or turnover intentions)	63, 64
	Task identity. The extent to which HCWs perceive their work as involving the completion of a whole and meaningful care task with a visible outcome Outcomes tied to the theme: health, job satisfaction, turnover intentions	Perceived completeness of work	Degree to which the job involves completing a whole and identifiable piece of work from beginning to end	60
**Job demands**	Safety hazards. Environmental and occupational risks faced by HCWs in clients’ homes or surrounding areas. Hazards included chemical, biological, physical, and ergonomic risks, often exacerbated by inadequate protections or unpredictable working environments Outcomes tied to the theme: emotional ill-being, injury, health problems, turnover intentions	Exposure to chemical, biological, and infectious agents	Contact with hazardous cleaning agents, drug residue, bodily fluids, or infectious diseases, often without adequate protective equipment or information	1, 17, 18, 23, 27, 30, 31, 46, 59, 65, 77
		Unsanitary and cluttered home environments	Presence of clutter, poor hygiene, and unsafe home layouts, creating physical and psychological hazards	9, 10, 30, 33, 45, 46, 47, 52, 67, 74, 75, 77
		Exposure to pests and infestations	Contact with bedbugs, lice, cockroaches, and other infestations affecting hygiene and health	1, 18, 27, 45, 46, 59, 65, 67, 77
		Sharps and medical waste	Presence of improperly disposed medical sharps or malfunctioning equipment posing risk of injury or infection	31, 45, 46, 47, 59
		Pets and animal-related risks	Injuries and strain caused by uncontrolled or aggressive pets in the client’s home	10, 17, 27, 46, 47, 65, 67, 77
		Fire, smoke, and oxygen-related hazards	Fire risks caused by clients’ behaviors (e.g., smoking indoors, cooking forgetfulness) and environmental conditions	1, 17, 27, 46, 47, 59, 65, 73, 77
		Slips, trips, and falls (STFs)	Environmental hazards (e.g., slippery floors, rugs, poor lighting) that cause STFs and musculoskeletal injuries	52, 59, 65, 77
		Neighborhood and home safety	Safety concerns related to clients’ neighborhoods (e.g., crime, poor lighting) or presence of weapons in the home	18, 45, 46, 47, 74
		Air quality and temperature conditions	Hazardous or uncomfortable indoor air conditions, including poor ventilation, extreme heat or cold, smoke, and humidity	18, 31, 46, 77
		Travel-related hazards	Physical or emotional risks related to transportation requirements, including vehicle accidents, unsafe travel modes, or long commutes	10, 30, 40, 45, 46
	Workload pressures. Excessive or increasing demands placed on HCWs that strain their time, energy, and emotional or physical capacity. This includes high task volume, time pressure, overwork, and demands that go beyond formal duties. Outcomes tied to the theme: physical and psychological ill-being, stress, turnover, and care quality	Quantitative demands	High volume or intensity of tasks to be completed within limited time, including both direct and indirect care duties, often exacerbated by client complexity and systemic constraints	2, 3, 6, 7, 19, 20, 21, 33, 37, 39, 50, 52, 61, 68, 74
		Time pressure	Insufficient time to complete tasks adequately, leading to stress, fatigue, missed care, and coping strategies like skipping breaks or rushing visits	2, 3, 7, 19, 21, 28, 30, 41, 45, 46, 53, 57, 60, 63
		Overwork	Regularly working beyond paid hours or capacity, including unpaid overtime, extended on-call availability, or physical strain due to long shifts	17, 20, 34, 39, 46, 69, 74
		Illegitimate tasks	Requests or expectations to perform tasks outside formal job responsibilities, often due to client or family demands, blurring boundaries and increasing strain and risk for injury	5, 12, 22, 24, 31, 34, 46, 53, 59, 65, 73
		Technology-related demands	Use of technological tools (e.g., smartphones, apps) in care delivery, which may enhance efficiency or professionalism but also contribute to additional workload, impersonal interactions, or frustration (especially when duplicative or difficult to use)	53, 74, 79
	Emotional demands. Emotional challenges encountered by HCWs arising from the interpersonal, psychological, and social nature of care work. These include internal strain from emotional involvement with clients, and external stressors such as violence, discrimination, and dealing with death or family conflict. Outcomes tied to the theme: mental exhaustion, stress, depression, sleep problems, job satisfaction, turnover, burnout	Emotional strain related to care work	Emotional burden due to caregiving responsibilities, including emotional closeness, role conflict, or navigating complex family dynamics	7, 18, 40, 41, 44, 49, 56, 57, 67, 71
		Dealing with client death and dying	Emotional reactions and grief experienced when clients decline or pass away	9, 10, 11, 12, 15, 17, 34, 42, 45, 50, 67, 71, 79
		Demands for hiding emotions	Expectations that HCWs suppress or regulate emotional expressions during interactions with clients, often resulting in emotional strain	17, 34, 57, 75
		Exposure to violence	Direct or indirect experiences of verbal, physical, or sexual violence from clients, families, or others, either firsthand or as witnesses	1, 4, 5, 7, 10, 12, 15, 17, 18, 24, 26, 27, 30, 31, 34, 35, 39, 42, 46, 56, 59, 61, 65, 67, 79
		Exposure to discrimination	Experiences of racism, xenophobia, or other forms of discrimination linked to HCWs’ race, ethnicity, gender, or migration status	5, 20, 28, 34, 46, 58, 59
	Physical demands. The physical effort required to perform HBC tasks, including manual handling of clients, mobility-related tasks, domestic labor, and commuting between homes Outcomes tied to the theme: musculoskeletal pain, stress, injury, fatigue, mental exhaustion, sleep problems, turnover, job satisfaction	Client handling and mobility support	Demands related to lifting, transferring, and physically assisting clients, often in ergonomically challenging environments	9, 10, 20, 27, 30, 31, 33, 42, 44, 45, 46, 47, 49, 52, 59, 65, 72, 73
		Domestic and task-related physical labor	Physical exertion associated with household chores (e.g., cleaning) or physical support with activities of daily living or equipment	1, 27, 46, 52
		Physical demands and worker health	Perceived physical intensity of the job and its impact on workers’ physical and mental health, including injury, fatigue, and sleep problems	7, 16, 17, 18, 34, 37, 40, 41, 43, 50, 66, 69
	Uncertainty. The unpredictability and lack of information, particularly in relation to client behaviours, unknown or complex medical conditions, and type of tasks they have to perform Outcomes tied to the theme: stress	Situational unpredictability	Unexpected or rapidly changing circumstances in the care setting that demand flexibility and quick judgment	9, 67
		Attending unfamiliar clients	Being assigned to clients without prior knowledge or relationship	45, 50
		Uncertainty about client medical conditions	Lack of information or preparation regarding clients’ medical, physical, or psychological conditions	34, 67, 71
**Relational resources**	Supportive leadership. Perceptions of supervisors’ presence, availability, and fairness, encompassing their ability to provide practical assistance, emotional support, recognition, and clear communication of boundaries. This also includes oversight of work tasks, responsiveness in crises, and respectful, fair treatment. Outcomes tied to the theme: job satisfaction, stress, intention to remain employed, professional efficacy and cynicism (burnout dimensions), emotional well-being	Supervisor support and responsiveness	Availability, approachability, and provision of practical or emotional assistance from supervisors or case managers when workers face challenges, emergencies, or need to set boundaries with clients	16, 18, 21, 22, 35, 46, 52, 57, 65, 71, 73, 74, 75
		Feedback and recognition	Provision of constructive feedback, praise, and acknowledgment of workers’ performance or contributions	7, 29, 76
		Supervision and oversight	Managerial monitoring and guidance of task execution, particularly when duties are expanded, shifted, or delegated, to ensure quality and safety of care	3, 19, 32, 55, 60, 68
		Relational justice	Perceived fairness, respect, and attentiveness from supervisors, including consideration of workers’ perspectives when making decisions and during daily interactions	21, 63
	Coworker support. The ways in which support from peers and other professionals (within or across teams) affects HCWs’ ability to manage job demands, maintain well-being, and deliver quality care Outcomes tied to the theme: emotional well-being, stress, retention	Emotional support and coping	Emotional reassurance, camaraderie, or shared experiences that help HCWs manage stress, emotional strain, or demanding situations	9, 18, 22, 25, 29, 57, 69, 75
		Instrumental peer support	Task-related assistance or practical help that enables safe, effective, or efficient care	2, 9, 31, 33, 34, 73, 79
		Teamwork and self-organized teams	Team-based approaches, with or without formal leadership, that influence workload distribution, job satisfaction, and turnover intentions	8, 9, 54, 63, 64, 75
		Support from other professionals	Supportive interactions with non-HCW colleagues (e.g., nurses, physicians) that influence skill development and a sense of recognition (when they took into account their input)	15, 71, 74
		Working alone	The stress, risks, and feelings of isolation associated with having no coworkers present during home visits or emergencies	9, 30, 31, 44, 45, 50, 63, 69, 73, 74
		General peer support and limitations	Broader references to general definitions of peer support and related outcomes	7, 60, 68, 74
	Client–carer relationship. Interpersonal dynamics between HCWs, their clients, and clients’ families, including appreciation, support, trust, closeness, and boundary-setting Outcomes tied to the theme: emotional well-being, job satisfaction/motivation, quality of care, turnover	Appreciation and support	Expressions (or absence) of gratitude, respect, and understanding from clients or their families, which can enhance HCWs’ emotional well-being and job satisfaction, or when lacking, contribute to feelings of undervaluation and turnover intentions	9, 12, 15, 18, 21, 22, 29, 31, 34, 46, 49, 71
		Quality and closeness of relationship	The degree of relational closeness between HCWs and clients/families, ranging from distant to family-like, and its implications for job satisfaction, emotional strain, professional boundaries, and care delivery quality. This includes the benefits of trust and rapport, as well as the potential stressors of over-attachment or strained relationships	5, 8, 11, 12, 14, 15, 18, 19, 22, 29, 30, 31, 32, 34, 42, 44, 46, 53, 62, 71, 72, 73, 74, 75, 79
	Non-work social support. This theme captures emotional and psychological support that HCWs receive from individuals or networks outside their workplace, including family, friends, pets, religious communities, and transnational connections. These forms of support contribute to coping with high demands Outcomes tied to the theme: emotional well-being	Emotional support from family and friends	Emotional support provided by family members or friends, often used to process work stress or build resilience	5, 17, 22, 34, 50
		Absence of social support and its consequences	Lack of access to non-work social networks, particularly for migrant HCWs, contributing to stress, loneliness, or homesickness	13, 42
		Alternative sources of support and belonging	Support derived from non-traditional or community-based sources, such as religious beliefs, pets, or virtual and collective networks	22, 34, 56
**Relational demands**	Conflict. Interpersonal tensions, disagreements, or problematic behaviors that may arise with clients, clients’ families, other professionals, or agency staff. Outcomes tied to the theme: emotional strain (stress), turnover, job satisfaction, general health	Difficult clients	Challenges stemming from clients’ behaviours, attitudes, or medical conditions that are stressful	5, 9, 11, 15, 17, 18, 22, 30, 31, 33, 34, 35, 44, 47, 49, 71, 75, 78, 79
		Relational conflicts	Disagreements, tensions, or strained relationships with clients, their families, other professionals, or agencies, that can escalate to violence, and that contribute to stress or turnover	15, 31, 33, 37, 60, 67
**Personal resources**	Job meaning. The personal significance HCWs attribute to their work, encompassing intrinsic and extrinsic motivation, relational value, contribution to clients’ well-being, and opportunities for personal or professional growth. Outcomes tied to the theme: job satisfaction (rewarding) retention	Intrinsic meaning and contribution to others	Finding value and fulfilment in helping clients, improving their quality of life, and making a difference. Often associated with retention, job satisfaction, and motivation despite challenges	12, 15, 21, 22, 28, 31, 34, 35, 42, 45, 46, 49, 50, 53, 59, 71, 74, 75
		Financial motivation	Viewing the job primarily as a source of income or security, sometimes alongside intrinsic motivations	15, 34, 42, 53, 78
		Self-improvement	Seeing the role as an avenue for personal growth, skill acquisition, and self-fulfilment	15, 49, 50
	Resilience and coping. Individual strategies, attitudes, and behaviors that enable HCWs to manage stressors, and protect against emotional strain in challenging work environments Outcomes tied to the theme: emotional well-being, stress	Emotional self-regulation and positive attitude	Strategies to manage personal emotions, maintain optimism, and prevent emotional exhaustion through mindset and healthy detachment	2, 29, 34, 50
		Self-determination and boundary setting	Asserting control over working conditions, expressing needs, and refusing inappropriate or unauthorized demands	34, 60
	Self-efficacy. HCWs’ belief in their ability to successfully perform job tasks and manage work-related challenges, which is shaped by training, preparedness, available resources, and experience Outcomes tied to the theme: job satisfaction, performance, physical exertion, stress, burnout	Self-efficacy linked to training and preparedness	The influence of prior training, skill development, and preparedness on confidence and perceived competence	13, 21, 51, 70
		Self-efficacy linked to safety and working conditions	Associations between perceived self-efficacy and factors such as safety climate, and availability of job resources	7, 26, 36, 37, 76
		Self-efficacy and job meaning/satisfaction	How perceptions of competence or adequacy influence intrinsic motivation and job meaning	35, 72
	Legal awareness. HCWs’ understanding of, and ability to navigate, the legal frameworks, rights, and procedures that affect their immigration status, employment, and protection from abuse Outcomes tied to the theme: labor exploitation	Legal awareness and immigration/work permit requirements	Knowledge of immigration or work permit requirements, and how bureaucratic processes affect employment stability	15
		Impact of legal knowledge on decision-making and employment relationships	How awareness (or lack thereof) of legal rights shapes HCWs’ employment decisions, working conditions, and relationships with employers	15
		Legal awareness as protection from exploitation or abuse	Understanding legal rights as a safeguard against exploitation, abuse, or unsafe working conditions	4, 34
	Skill and knowledge. The cognitive, technical, and problem-solving abilities that enable HCWs to perform their work effectively, respond to complex or unpredictable situations, maintain quality of care, and protect themselves and clients from harm Outcomes tied to the theme: stress, falls, injury, job satisfaction	Complex case management	Applying professional competence, critical thinking, and adaptability to manage complex client situations or high-stakes care needs	8, 46, 50, 59, 73, 77, 79
		Crisis anticipation and response	Anticipating, preventing, and responding to predictable or sudden crises, sometimes adapting or going beyond standard protocols to protect safety and quality of care	67, 73
		Handling qualitative demands	Managing cognitive demands such as unpredictability, complex decision-making, and high responsibility inherent to skilled care work	37, 41
		Task focus and attention	Sustaining concentration and accuracy while minimizing errors or accidents, even in environments with frequent distractions or interruptions	47, 52, 63
	Proactive behavior. Actions initiated by HCWs on their own accord and beyond formal job requirements to improve care delivery, manage workload, and enhance safety. These behaviors function as self-initiated strategies that build job resources, mitigate risks, and support well-being in demanding work environments Outcomes tied to the theme: emotional well-being, injury	Self-care	Deliberate activities undertaken outside work tasks to preserve emotional health and prevent strain, including leisure, relaxation, and social engagement	29, 34
		Skill development	Voluntary efforts to expand professional knowledge and abilities outside mandated training, enhancing competence	10, 73
		Task prioritization	Independently organizing, sequencing, or adapting tasks based on client needs, preferences, or situational demands to manage workload and time pressure	2, 3, 29
		Personal safety behaviors	Self-directed actions to safeguard personal health and prevent injury, including hazard prevention, and adaptive safety strategies	10, 37, 44, 46, 52, 58, 67

*Note*. HCWs = home care workers. “Study Nº” refers to the identifiers assigned to the 79 included articles, which can be matched to each article using the first column (“Nº”) of Supplementary Material, File 3. The number of studies reported in the table reflects all articles in which each theme or code appeared. This may include more studies than those cited in the main text, as more salient findings or illustrative references were sometimes prioritized to maintain clarity and avoid overloading the text with citations.

#### Well-being outcomes

All well-being outcomes identified during the thematic analysis were grouped into 17 codes. The nine negative well-being or ill-being codes were grouped into two broader themes: (a) psychological and occupational ill-being, and (b) physical ill health. Similarly, the positive well-being outcomes (*N* = 8) were grouped into two themes: (a) psychological and physical well-being for variables that included a combination of the two indicators and (b) psychological and occupational well-being. In addition, two secondary work-related outcomes frequently emerged in relation to the well-being of HCWs: quality of care and work–home spillover or imbalance.

##### Ill-being outcomes

Negative well-being outcomes were grouped into two categories: psychological ill-being and physical ill-health. Psychological ill-being included emotional distress, such as frustration, sadness, and homesickness (e.g., Andersen & Westgaard, 2015; Cacciapuoti et al., 2025); burnout and emotional exhaustion (e.g., Bakker et al., 2003; Hanson et al., 2015; Rong el al., 2022); depressive symptoms and suicidality (e.g., Ayalon, 2012; Pinto et al., 2022); turnover intentions (e.g., Butler et al., 2010; Lee & Oh, 2023; Rahnfeld et al., 2016), and stress (e.g., Ruotsalainen et al., 2020), anxiety (e.g., Fernández-Carrasco et al., 2022), and occupational strain, including job strain and effort-reward imbalance (e.g., Larsson et al., 2013; Ruotsalainen et al., 2023; Simon et al., 2008). Physical ill-health outcomes included musculoskeletal disorders, pain, and work-related injury (e.g., Lee & Jang, 2016; Lindholm et al., 2022); general physical health problems (e.g., respiratory, cardiovascular and dermatological problems (Agbonifo et al., 2017) and illness-related consequences such as sick leave and sickness presenteeism (e.g., Aronsson et al., 2014); sleep disturbance (e.g., Hanson et al., 2015; Lindholm et al., 2022); and physical exhaustion (e.g., Strandell, 2020).

##### Positive well-being outcomes

The outcomes of positive well-being were mostly related to psychological well-being. Job satisfaction was the most frequently identified positive outcome (e.g., Ravenswood et al., 2017; Rong et al., 2022; Ruotsalainen et al., 2020, 2023), alongside emotional well-being and happiness (e.g., Larsson et al., 2013; Minguela-Recover et al., 2022); life satisfaction (e.g., Pinto et al., 2022); retention and occupational attraction, including reasons to stay and intention to remain employed (e.g., Gusoff et al., 2025; Kelly et al., 2024; Tourangeau et al., 2014); and work engagement and thriving (e.g., Chang et al., 2025; Nielsen & Jørgensen, 2016). Wider positive indicators of well-being also emerged, involving various aspects of well-being (e.g., psychological, physical, and social), such as recovery (Aronsson et al., 2014), quality of life (Fernández-Carrasco et al., 2022), and work ability (Larsson et al., 2012, 2013).

##### Secondary work-related outcomes

Two additional work-related outcomes frequently emerged in relation to job demands and resources and the well-being of HCWs. First, aspects related to the quality of provided care (e.g., Bagnasco et al., 2024; Ruotsalainen et al., 2020). Second, work–home spillover, mostly in the direction of how work interfered with workers’ private and family lives (e.g., Franzosa et al., 2019; Martínez-de la Torre et al., 2025).

#### Macro resources

##### Occupational esteem

The theme refers to the social value and professional recognition attributed to the HBC work. Participants reported having a low occupational status and their work being frequently undervalued. Personal care workers described being treated as less important than other, more clinical, health care professionals (e.g., nurses) and being excluded from multidisciplinary communication or dismissed by clients and their families (Kusmaul et al., 2020; Markkanen et al., 2014; Sterling et al., 2018). Some studies have noted that personal care workers are referred to as “maids,” “chore workers,” or “glorified housekeepers,” rather than as care professionals (Franzosa et al., 2019; Markkanen et al., 2014; Martínez-de la Torre et al., 2025; Schoenfisch et al., 2017). These experiences reflect a broader societal perception of HBC work as low-skilled and of low status. Personal care workers also described the structural consequences of this perception. Live-in migrant workers reported being professionally and legally vulnerable because of their lower occupational status, dependence on clients and families, and limited knowledge of the legal system (Ayalon, 2009). Other participants reported being ignored or undervalued by agency staff, clients, and the public (Sterling et al., 2018; Zoeckler, 2018). Although the participants identified themselves as skilled and providing essential work, they perceived a discrepancy between their actual contributions and how their role was regarded, despite the increasing delegation of tasks and responsibilities (Barken et al., 2015; Schoenfisch et al., 2017). Perceived low occupational esteem was also linked to workforce shortages, as poor public image contributed to recruitment difficulties (Ruotsalainen et al., 2020).

##### Unionization

Unionization emerged as a potential resource, particularly for personal care workers. Those who were unionized reported receiving instrumental support, such as training opportunities, access to food banks, and backing when pay checks were delayed (Delp & Muntaner, 2010). Unions also fostered a sense of collective identity and dignity, finally making workers feel visible. The companionship fostered within the union spaces gave workers the confidence to advocate for themselves. Union membership has also been related to greater job satisfaction (Delp & Muntaner, 2010), whereas its absence among HBC nurses has been tied to reduced intention to remain in the job (Tourangeau et al., 2014). In client-hired arrangements, where formal organizational protections are lacking, union representation could fill that gap by providing structural support (Karlsson et al., 2020). However, unionization and collective bargaining remain limited across the sector. Contract violations and fraudulent practices have been reported, reflecting the vulnerability of these unregulated working environments (Martínez-Buján & Moré, 2025).

#### Organizational resources

##### Employer support

Employer support practices promote a sense of value and safety. It was tied to work satisfaction (Hsu & Chen, 2025), well-being (Martínez-de la Torre et al., 2025), and less turnover intentions (Lee & Jang, 2016). Employers showed support in different ways. HCWs valued an agency contact who listened, was available, and supported their emotional well-being (Janssen & Abbott, 2023; Kusmaul et al., 2020). Instrumental support, such as adjusting schedules or receiving support when working with difficult clients, was also appreciated (Butler et al., 2012; Denton et al., 2002). Support during crises, especially in cases of false accusations or client conflict, was described as a protective practice (Butler et al., 2010). Employer responsiveness to concerns about safety and disrespect made HCWs feel taken seriously (Quinn et al., 2016), unsupported, and less safe (Jepsen et al., 2025). Employers also supported HCWs by managing client expectations through role definitions, clear care plans, and communicating them to clients. This support helped prevent conflict when refusing tasks beyond job duties by redirecting clients to the agency or the care plan (Karlsson et al., 2020; Minguela-Recover et al., 2022).

##### Safety climate

Safety climate perceptions were linked to higher participation in risk management, perceived self-efficacy, and work ability (i.e., perceived ability to work considering their health) (Larsson et al., 2012, 2013). However, client-hired HCWs described poorer safety climates, feeling that their concerns were taken less seriously (Quinn et al., 2016).

##### Employment benefits

Home care workers reported a lack of basic employment benefits, such as paid leave, retirement contributions, and reimbursement for travel or car-related expenses (Green & Ayalon, 2018; Markkanen et al., 2014; Pinto et al., 2022; Zhang et al., 2022). This absence has been linked to turnover intentions (Butler et al., 2010; Tourangeau et al., 2014). The lack of fair compensation for greater responsibilities has also been reported (Barken et al., 2015). In contrast, other job-related rewards, such as performance bonuses and recognition programs, were perceived as supportive and contributed to emotional well-being (Janssen & Abbott, 2023; Kusmaul et al., 2020). Access to health care benefits emerged as particularly important in contexts where coverage depends on employment status (e.g., USA). A lack of insurance has been linked to turnover intentions (Butler et al., 2010, 2012), whereas adequate health care coverage facilitated job satisfaction and retention (Kusmaul et al., 2020; Rong et al., 2022).

##### Work equipment

Limited access to the necessary equipment for safe care provision was common, although its availability was linked to positive emotions at work (Van de Weerdt & Baratta, 2015). The absence of mechanical lifts and other assistive devices resulted in musculoskeletal disorders and increased injury risk during transfer tasks (Markkanen et al., 2014, 2017; Sims-Gould et al., 2013). It also prevented workers from adhering to safety regulations (Larsson et al., 2013). Access to protective gear and medical supplies was inconsistent. While some workers reported being provided with the necessary materials (Aronsson et al., 2014; Kusmaul et al., 2020; Zoeckler, 2018), others had to purchase their own supplies (Tourangeau et al., 2014; Zhang et al., 2022). This added financial burden caused frustration and influenced turnover intentions (Tourangeau et al., 2014).

##### Service coordination

Effective planning, scheduling, and communication management practices influenced the well-being of agency-hired HCWs. Good planning helped improve work–life balance (Minguela-Recover et al., 2022). However, most findings reported challenges. Centralized scheduling excluded HCWs’ input, resulting in unrealistic routines, increased travel, and limited time spent with clients (Ruotsalainen et al., 2020). Poor internal communication led to mental strain and turnover intentions (Butler et al., 2010; Zoeckler, 2018), while receiving information relevant to adequate job performance was linked to job satisfaction (Cindrić & Malnar, 2025). Home care workers also lacked sufficient information about clients’ health conditions (Denton et al., 2002; Sterling et al., 2018). Coordination across health care settings was also limited, particularly during transitions from hospital to home care, which disrupted continuity (Larsson et al., 2013). Despite being central to clients’ daily care, HCWs were often excluded from care planning conversations (Fleming & Taylor, 2007; Sterling et al., 2018). When the emotional strain was caused by poor or inefficient management rather than the care tasks themselves, HCWs were more frustrated and less willing to tolerate these structural issues (Van de Weerdt & Baratta, 2015).

##### Hiring practices

One study reported that ineffective recruitment and onboarding resulted in frustration, burnout, and intentions to leave the company, as new hires were unprepared for the demands of HBC work and unaware of what the job entailed (Zoeckler, 2018).

##### Training

Home care workers valued high-quality in-service training as it supported their safety, well-being, and retention (Larsson et al., 2013; Ravenswood et al., 2017; Tourangeau et al., 2014; Zoeckler, 2018). However, such training was often inadequate or delayed (Brulin et al., 2000; Butler, 2018; Schoenfisch et al., 2017). Local workers emphasized the need for client-specific training (e.g., disease-specific) (Sterling et al., 2018), whereas migrant live-in HCWs lacked training on legal rights and protections (Chowdhury & Gutman, 2012; Kriegsmann-Rabe et al., 2023). Formal vocational training improved confidence, skill acquisition, and job satisfaction (Barken et al., 2015; Fleming & Taylor, 2007). In contrast, client-hired HCWs had fewer qualifications than those hired through agencies (Quinn et al., 2016). Mentoring and training on the job were also mentioned. Peer mentoring supported onboarding (Swedberg et al., 2013), but was often absent (Kusmaul et al., 2020). Experienced staff faced pressure to train new hires under tight schedules, which sometimes contributed to turnover (Tourangeau et al., 2014).

##### Career development

Although greater development opportunities were linked to lower burnout (Bakker et al., 2003), HCWs often reported limited chances for promotion or professional growth within HBC settings (Chowdhury & Gutman, 2012; Quinn et al., 2016; Zhang et al., 2022).

#### Organizational demands

##### Organizational change

Organizational restructuring in agency-based HBC often led to heavy workloads, job insecurity, time pressure, and stress, especially when it was introduced without adequate support (Andersen & Westgaard, 2015; Nielsen & Jørgensen, 2016). Employees perceived that cost-efficiency measures are prioritized at the expense of compassionate quality care (Andersen & Westgaard, 2015). Some studies have reported the disregard for relational care. Home care workers were expected to prioritize technical, measurable tasks over emotional, interpersonal care (Andersen & Westgaard, 2015; Van de Weerdt & Baratta, 2015). Some managers deemed the latter were either optional or irrelevant and excluded it from the time allocation. This discrepancy in perceptions of the role of a carer undermined workers’ sense of professional purpose and caused emotional distress. Staff shortages and an increasing caseload, often resulting from cutbacks, further intensified workloads and time constraints (Brulin et al., 2000; Larsson et al., 2013; Strandell, 2020). This led to psychological distress and impaired the quality of care provided.

Beyond increasing workload and time pressure, organizational change appeared to reshape the content of care itself. In some studies, relational and emotional aspects of care were treated as secondary to technical and measurable tasks, and time allocation practices left limited space for meaningful interaction with clients. This pattern is consistent with findings in other sections of the synthesis showing that positive client–carer relationships supported well-being and motivation, while time pressure hindered relationship-building.

##### Precarious employment

Across all employment settings, low and unstable incomes were a consistent source of strain. Added financial strain prevented workers from prioritizing their health and taking days off to recover when needed (Parella et al., 2024; Solano-Kamaiko et al., 2025). Irregular working hours intensified these challenges. Unpredictable and fluctuating schedules resulted in stress, mental exhaustion, lack of recovery, and work–life conflict (Bakker et al., 2003; Denton et al., 2002; Fleming & Taylor, 2007; Franzosa et al., 2019; Martínez-de la Torre et al., 2025; Solano-Kamaiko et al., 2025; Zoeckler, 2018). Precarious or exploitative employment conditions, such as part-time and casual contracts, were also reported and were linked to poorer well-being and turnover (Pinto et al., 2022; Tourangeau et al., 2014). Vulnerability was particularly pronounced among migrant live-in and client-hired workers, who reported poor contract terms (e.g., lack of fixed hours and protection from dismissal and subsequent loss of housing) (Cacciapuoti et al., 2025; Martínez-Buján & Moré, 2025; Parella et al., 2024). However, the low pay experienced by migrant HCWs had a dual meaning: it was low in their host country, but high in their home country, where they could support their family members by sending them money (Chowdhury & Gutman, 2012; Kriegsmann-Rabe et al., 2023).

Intermediary agencies for direct-hired workers sometimes helped formalize employment but offered limited protection (Martínez-Buján & Moré, 2025). Agency-hired HCWs generally had more structured and secure employment (Kelly et al., 2024; Quinn et al., 2016). In contrast, worker cooperatives were linked to better pay and job stability (Gusoff et al., 2025).

#### Job resources

##### Autonomy

Autonomy was described as the ability to make decisions, prioritize tasks, and exercise professional judgment. It was linked to job satisfaction, meaningfulness, and lower levels of burnout (Bakker et al., 2003; Cindrić & Malnar, 2025; Kusmaul et al., 2020; Ruotsalainen et al., 2020). Differences in autonomy were reported based on gender, ethnicity, role, and employment model. For example, nurses (Oomkens et al., 2016) and HCWs employed in worker cooperatives (Gusoff et al., 2025) reported greater autonomy. These cooperatives had more influence over patient care and agency-wide policies. However, financial insecurity at times restricted workers’ ability to exercise this resource (e.g., avoiding dangerous clients) (Butler, 2018). It was also constrained by a lack of control over the working environment and planning and discharge decisions, causing emotional strain (Brulin et al., 2000; Denton et al., 2002; Swedberg et al., 2013; Tourangeau et al., 2014).

##### Flexibility

Flexibility in scheduling was a rewarding aspect that had substantial consequences over job satisfaction and retention (Butler et al., 2012; Karlsson et al., 2020; Quinn et al., 2016). Employees appreciated employer practices regarding flexibility (i.e., allowing shift preferences to balance personal life responsibilities) (Markkanen et al., 2007; Minguela-Recover et al., 2022). However, flexibility also had a negative side, as HCWs were expected to adapt to the changing needs of clients and unpredictable care demands (Denton et al., 2015; Sims-Gould et al., 2013). Schedule control occasionally contributed to overwork, as employees assumed additional responsibilities beyond their regular duties (Markkanen et al., 2014). For migrant live-in HCWs, it created a sense of constant availability that impaired recovery from work (Parella et al., 2024).

##### Task variety

Task variety was linked to motivation and satisfaction. Diverse tasks and client needs were seen as rewarding and promoted retention (Brulin et al., 2000; Tourangeau et al., 2014), whereas repetitive work was related to lower satisfaction and increased stress (Denton et al., 2002).

##### Skill discretion

Skill discretion was described as both: a job requirement and a potential resource. The role demanded advanced skills and knowledge, particularly for monitoring clients’ conditions (Barken et al., 2015; Larsson et al., 2013). Although it led to lower cynicism and higher professional efficacy among personal care workers (Bakker et al., 2003), no consistent links with well-being outcomes were found for nursing staff (Ruotsalainen et al., 2020, 2023).

##### Task identity

One study linked task identity (i.e., completing a meaningful piece of work) to better health and job satisfaction. It also buffered the effects of social conflict and indirectly reduced turnover intentions by increasing satisfaction (Rahnfeld et al., 2016).

#### Job demands

##### Safety hazards

HCWs faced numerous occupational hazards. They were frequently exposed to dangerous substances, including cleaning chemicals, drug residue, infectious agents, and bodily fluids, often without protective equipment (Agbonifo et al., 2017; Ghoroubi et al., 2023; Hittle et al., 2016; Karlsson et al., 2020). Client-hired aides reported more chemical and biological exposures (Quinn et al., 2016). Unsanitary or cluttered environments interfered with the delivery of care and contributed to slips, trips, and falls, emotional strain, and intentions to leave (Koivula et al., 2016; Tourangeau et al., 2014; Van de Weerdt & Baratta, 2015). Pest infestations, including bedbugs and cockroaches, have been frequently reported (Hittle et al., 2016; Schoenfisch et al., 2017). Sharps injuries have been linked to improper disposal and malfunction (Markkanen et al., 2017), while bites and scratches from aggressive pets posed an additional health risk (Butler, 2018; Schoenfisch et al., 2017). Fire and smoke-related hazards included clients smoking indoors or while on oxygen and forgetting to turn off cooking appliances (Markkanen et al., 2014; Wills et al., 2016). Second-hand smoke exposure was common and led to respiratory symptoms (Delp & Muntaner, 2010; Swedberg et al., 2013). During home visits, HCWs had to visit neighborhoods with poor lighting, high crime rates, and firearms (Denton et al., 2002; Markkanen et al., 2014). Poor indoor air quality and high temperatures have also been reported to be harmful (Karlsson et al., 2020; Wills et al., 2016). Transportation posed additional safety risks (e.g., car–scooter accidents) (Butler, 2018; Jepsen et al., 2025; Liaset et al., 2024).

##### Workload pressures

Significant workload pressures tied to high task volume, time constraints, and expectations to work beyond one’s formal duties were identified. Excessive workload volume and intensity (coded in the synthesis as “quantitative demands”) were common and were associated with stress, poorer quality of life, and reduced quality of care (Bagnasco et al., 2024; Fernández-Carrasco et al., 2022; Sjöberg et al., 2020). Limited time for visits and increased indirect tasks led to stress, exhaustion, and compensatory behaviors, such as skipping breaks or lowering care standards (Aronsson et al., 2014; Bakker et al., 2003; Lindholm et al., 2021). Migrant live-in workers were particularly vulnerable, often required to be available 24/7 (Kriegsmann-Rabe et al., 2023). Overwork, including unpaid overtime and long shifts, has been linked to stress, anxiety, and turnover intentions (Fernández-Carrasco et al., 2022; Lee & Oh, 2023; Tourangeau et al., 2014). Illegitimate tasks (i.e., performing duties outside of one’s job description) were a common source of added workload and were perceived in a negative way when they seemed to come from the arbitrary wishes of the clients (Karlsson et al., 2020). Technology-related demands had mixed effects. While digital tools improved efficiency and professionalism, they also increased workload (Nielsen & Jørgensen, 2016; Tourangeau et al., 2014; Zoeckler, 2018).

##### Emotional demands

Frequently reported emotional demands were shaped by interpersonal relationships, close client relationships, and exposure to distressing environments. These demands contributed to mental strain, especially when workers were caught up in family disputes (Sims-Gould et al., 2013; Sterling et al., 2018). Tending to terminally ill or vulnerable patients was particularly challenging for HCWs who developed close ties with their clients (Butler et al., 2012; Markkanen et al., 2007; Zoeckler, 2018). Concerns of job loss following a client’s death have exacerbated this strain (Sterling et al., 2018). The expectation that workers should remain emotionally composed added to the emotional load (Kriegsmann-Rabe et al., 2023; Van de Weerdt & Baratta, 2015). Verbal, physical, and sexual violence were frequently reported, often in dementia care, and were linked to stress, depression, and job dissatisfaction (Butler, 2018; Denton et al., 2002; Hanson et al., 2015; Jepsen et al., 2025). The refusal of illegitimate task requests was another trigger for violence (Karlsson et al., 2020). Workers also faced second-hand abuse (e.g., witnessing neglect) (Markkanen et al., 2014). Discrimination also resulted in emotional distress and poorer quality of life, particularly among migrant and racialized workers (Fernández-Carrasco et al., 2022; Markkanen et al., 2014; Pinto et al., 2022). However, culturally sensitive workplaces were associated with greater work satisfaction (Hsu & Chen, 2025).

##### Physical demands

Physical demands were prominent in HBC, which included client lifting, mobility assistance, and strenuous domestic tasks. These were linked to MSDs, sleep problems, and mental exhaustion (Bakker et al., 2003; Delp & Muntaner, 2010; Lindholm et al., 2021; Quinn et al., 2016). Transfer tasks have been reported as a safety concern (Jepsen et al., 2025; Karlsson et al., 2020). Walking between visits was reported as exhausting rather than healthy exercise (Liaset et al., 2024; Solano-Kamaiko et al., 2025). Caring for clients with high activities of daily living needs increased physical effort, requiring prolonged standing and movement (Lohne et al., 2024). Personal care workers reported higher physical demands than the nursing staff (Hittle et al., 2016). Among personal care workers, those working in rural settings described more physical demands than their counterparts in urban settings, which was linked to less access to organizational support (Cindrić & Malnar, 2025).

##### Uncertainty

Home care workers experienced uncertainty due to unpredictable situations, unfamiliar clients, and lack of information about medical conditions. This unpredictability, particularly in the context of dementia or palliative care, resulted in distress (Brulin et al., 2000; Sims-Gould et al., 2013). Not being prepared for clients’ complex needs further increased the emotional burden (Kriegsmann-Rabe et al., 2023; Sterling et al., 2018).

#### Relational resources

##### Supportive leadership

Leadership qualities influenced job satisfaction and well-being, with workers valuing instrumental help, feedback, and support, especially during crises (e.g., clients’ death) and difficult clients (Cindrić & Malnar, 2025; Denton et al., 2002; Fleming & Taylor, 2007; Franzosa et al., 2019; Markkanen et al., 2014; Petersen & Melzer, 2026). Positive reinforcement promoted emotional well-being (Janssen & Abbott, 2023), whereas unfavorable supervisory feedback or uninvolved managers prompted turnover intentions (Tourangeau et al., 2014; Van Waeyenberg et al., 2015). However, time pressure hinders relationship-building between supervisors and staff (Van de Weerdt & Baratta, 2015). Agency-based roles generally provide a more structured oversight than direct-hire arrangements (Kelly et al., 2024).

##### Coworker support

Support from coworkers influenced the ability of HCWs to manage demands, protect their well-being, and ensure the quality of care. Informal meetings with peers provided emotional support and helped workers mitigate professional loneliness and cope with high demands and job stress (Brulin et al., 2000; Franzosa et al., 2019; Solano-Kamaiko et al., 2025; Van de Weerdt & Baratta, 2015). In particular, cooperatives fostered a sense of camaraderie and belonging (Gusoff et al., 2025). In addition, providing instrumental support (e.g., helping with demanding clients or heavy workloads) reduced strain (Andersen & Westgaard, 2015; Koivula et al., 2016), while collaborating with other health professionals improved the skills of migrant workers (Chowdhury & Gutman, 2012). Self-organizing teamwork was linked to higher job satisfaction and lower turnover (Ruotsalainen et al., 2020, 2023), although the sudden implementation of this model increased workload and conflict (Ollé-Espluga et al., 2024). However, working alone increased psychological distress, feelings of isolation, and safety risks (Jepsen et al., 2025; Ruotsalainen et al., 2020; Swedberg et al., 2013).

##### Client-carer relationship

Positive client–carer relationships were linked to emotional well-being, job satisfaction, and retention (Chang et al., 2025; Janssen & Abbott, 2023; Rong et al., 2022; Tourangeau et al., 2014). Appreciation and gratitude from clients and their families enhanced workers’ morale and motivation to go the extra mile (Karlsson et al., 2020; Kriegsmann-Rabe et al., 2023). Good relationships also made clients more cooperative, improving safety and effectiveness, but it could also increase risk (i.e., relying on the HCWs instead of a lifting device) (Love et al., 2017). However, time pressure hindered opportunities for relationship-building, particularly when a high instrumental workload left less time for relational tasks (Van de Weerdt & Baratta, 2015). Relationships often evolved into family-like, trusting, relationships. While rewarding and reported in Franzosa et al. (2019) as central to home care worker emotional well-being, such attachment also intensified grief after a client’s death (Butler et al., 2012), evoked guilt when refusing illegitimate tasks (Karlsson et al., 2020), and increased workload (Markkanen et al., 2014). Even when advised to maintain a professional distance, the nature of prolonged, positive relationships made this difficult, as clients often became like family (Karlsson et al., 2020). Achieving balance (i.e., remaining sufficiently close yet professionally distant) was seen as necessary but challenging (Swedberg et al., 2013).

##### Non-work social support

Non-work social support, including family, friends, pets, religious communities, and transnational networks, fostered emotional well-being and resilience. Home care workers often relied on relatives or friends to process work-related stress (Delp & Muntaner, 2010; Franzosa et al., 2019), which also protected against suicidal thoughts and depression (Ayalon, 2012). Lack of networks led to loneliness, stress, and homesickness, especially among migrants (Cacciapuoti et al., 2025). Alternative sources, such as spirituality or faith, pets, associations, and virtual networks, also offered coping and belonging, with migrant live-ins often using remote contact to regulate emotions and maintain connections (Franzosa et al., 2019; Kriegsmann-Rabe et al., 2023; Parella et al., 2024).

#### Relational demands

##### Conflict

Conflicts and strain arose from difficult or demanding clients and strained relationships with clients, families, or other professionals. Difficulties included uncooperativeness, a lack of trust, high-needs illnesses, and refusal to follow care plans, which contributed to stress (Franzosa et al., 2019; Jepsen et al., 2025; Kriegsmann-Rabe et al., 2023; Sterling et al., 2018). Relational conflicts involved hostility from clients’ families (Chowdhury & Gutman, 2012), disagreements over care plans (Sims-Gould et al., 2013), and disputes with peers or other health providers (Koivula et al., 2016; Sims-Gould et al., 2013). Conflicts could escalate to violence (Karlsson et al., 2020) and contribute to turnover and reduced job satisfaction (Rahnfeld et al., 2016).

#### Personal resources

##### Job meaning

Home care workers often found their job meaningful and rewarding because they feel they are helping improve their clients’ lives, despite the challenges that come with the job (Butler et al., 2012; Chowdhury & Gutman, 2012; Hsu & Chen, 2025; Karlsson et al., 2020; Kusmaul et al., 2020). The joy of caring for others and making a difference in their lives was a factor in retention (Quinn et al., 2016; Tourangeau et al., 2014). However, financial reasons were the main motivation for some (Lindquist et al., 2012; Zhang et al., 2022). The role also offered opportunities for self-improvement and stimulation, providing fulfilment through exposure to diverse experiences (Chowdhury & Gutman, 2012; Minguela-Recover et al., 2022).

##### Resilience and coping

Strategies for resilience and coping with exhaustion included regulating emotions, maintaining optimism, the ability to detach, and mindfulness (Andersen & Westgaard, 2015; Janssen & Abbott, 2023). Disconnecting from work supported well-being (Kriegsmann-Rabe et al., 2023; Minguela-Recover et al., 2022). Self-determination, including asserting desired conditions, saying “no” to rights violations and unauthorized tasks, were protective practices that fostered control over work situations (Kriegsmann-Rabe et al., 2023; Rahnfeld et al., 2016).

##### Self-efficacy

HCWs’ belief in their ability to perform their work effectively was associated with positive outcomes, including job satisfaction and reduced perceived physical strain, stress, and burnout (Hanson et al., 2015; Kusmaul et al., 2020; Stawnychy et al., 2023). Higher self-efficacy was tied to training (Fleming & Taylor, 2007; Stawnychy et al., 2023) and supportive safety environments (Larsson et al., 2012). Conversely, feelings of inadequacy arose with role uncertainty (Mitchell et al., 2023), when HCWs perceived that clients were not receiving sufficient care (Strandell, 2020), or when migrant HCWs felt unprepared for difficult tasks or had limited language skills (Cacciapuoti et al., 2025).

##### Legal awareness

Legal literacy was particularly relevant for migrant HCWs, who were vulnerable to exploitation due to limited legal knowledge and language barriers (Kriegsmann-Rabe et al., 2023). Some reported being misled by intermediary agencies regarding working conditions or immigration status. Navigating foreign legal systems was challenging due to delays in renewing required residency documents (Chowdhury & Gutman, 2012). In Israel, a lack of rights awareness left Filipino HCWs exposed to exploitation, compounded by mistrust and isolation (Ayalon, 2009). Participants valued receiving this information before or on arrival (Chowdhury & Gutman, 2012).

##### Skill and knowledge

Cognitive and problem-solving skills were necessary for managing complex cases, responding to crises, and dealing with unpredictability (Lindholm et al., 2021; Swedberg et al., 2013). Complexity was sometimes viewed as a challenge demand (i.e., stimulating) (Minguela-Recover et al., 2022). Such capabilities and focus were relevant for safety and quality of care, given that workers were subject to distractions and interruptions while providing care (Markkanen et al., 2017; Ruotsalainen et al., 2020).

##### Proactive behavior

Home care workers proactively protected their well-being, improved safety, and managed workload. Strategies included practicing self-care through leisure and social activities (Janssen & Abbott, 2023; Kriegsmann-Rabe et al., 2023), developing skills outside work hours (Butler, 2018; Swedberg et al., 2013), task prioritization based on client needs (Andersen & Westgaard, 2015), and adopting personal safety measures, such as preventing hazards or adapting rules for safer, more effective care (Muramatsu et al., 2018; Sims-Gould et al., 2013).

## Discussion

This article is, to our knowledge, the first MMSR integrating evidence on HCWs’ well-being across occupations, employment arrangements, and migration statuses using a dual theoretical framework (JD–R and W–HR). By going beyond fragmented topic-specific syntheses, this review provides a comprehensive framework of how multilevel demands and resources interact to shape the well-being of HBC workers. The findings reveal a consistent pattern: excessive quantitative and emotional demands, coupled with limited organizational resources, drive strain, whereas specific buffers (e.g., supervisor support, information flow, and positive relationships) foster motivation and mitigate harm. Notably, we emphasize mechanisms that have received little attention until now, such as the systematic devaluation of relational care and the essential role of rights awareness. Some effects were particularly pronounced among direct-hires and migrant HCWs, including poorer health and safety climates, a lack of organizational or legal protections, and worse contract terms. The type of occupation also shaped HCWs’ experiences: personal care workers reported lower professional esteem and fewer protective resources than nursing care workers. These moderating factors help explain the cross-study differences and guide the application of the framework to other LTC contexts.

The results align with JD–R’s proposition that high demands and limited resources increase job strain and with W–HR’s expectations that these conditions lead to conflict between work and private life. Our review not only confirms existing evidence on uncontrolled work settings, exposure to hazards, violence, isolation, the emotional impact of client death, and retention challenges (Clari et al., 2020; Findlay & Robertson, 2024; Forward et al., 2024; Grønoset Grasmo et al., 2021; Phoo & Reid, 2022; Thwaites et al., 2023; Zhong & Shorey, 2023), but also extends the field in three ways. First, by integrating the W–HR model to capture the influence of nonwork demands and resources. Second, by identifying the devaluation of relational care as a crucial mechanism that weakens motivation. Third, by identifying legal awareness and effective coordination as critical buffers, all of which point to where the interventions should be targeted.

### Structural constraints

One of the most salient findings is the extent to which precarious employment and legal insecurity act as chronic structural demands, limiting workers’ ability to recover and undermining their sense of control. These conditions align with the JD–R proposition of persistent health-impairing processes and W–HR spillover effects, as financial and legal vulnerability intensifies stress both at work and at home. Our review contextualizes these risks within the broader framework of poorly regulated care markets, where women and migrants are disproportionately vulnerable to exploitation and harassment (Fisher, 2021; Thwaites et al., 2023). These patterns mirror wider debates about gendered and racialized labor in the global care economy. However, this review extends the field by identifying legal literacy as a critical personal resource. Evidence shows that workers who know their rights are more empowered to resist exploitation. Therefore, reforms must include legal training and enforceable protections. We also found that brokering platforms often provide little post-placement support (Martínez-Buján & Moré, 2025), leaving client-hire workers without any structural backing. This is common in the context of the gig economy or marketized care (Kost et al., 2020).

### Job design issues

The findings also show how inadequate structural support can make everyday stressors more harmful (i.e., loss spirals). Weak supervisory responsiveness, exclusion from care planning, and fragmented coordination depleted key resources, leading to excessive workloads and uncertainty for HCWs. While this finding aligns with prior qualitative evidence on physically strenuous and poorly supported tasks (Grønoset Grasmo et al., 2021), our review advances the understanding of these issues by linking them to systemic patterns of attrition and compromised care quality. Autonomy and flexibility are generally viewed as resources. However, our evidence shows their ambivalent effects. Autonomy sometimes enables professional discretion, yet in practice it often translates into taking on illegitimate tasks or being permanently on call. Similarly, flexible scheduling can enhance work–life balance, but it can also blur boundaries and prevent recovery. These contradictions illustrate the necessity of designing organizational systems that provide flexibility and autonomy without worsening precarity. We also found that the requirement to use new digital tools and screens during visits acted either as a challenge or as a hindrance demand as it involved both positive and negative outcomes (Nielsen & Jørgensen, 2016; Tourangeau et al., 2014; Zoeckler, 2018).

### Uncontrolled working environment

Another critical contribution concerns the uncontrolled nature of the home setting, which exposes workers to safety hazards, violence, and uncertainty. Evidence indicates that unpredictable client health conditions, environmental risks, and travel hazards increase stress levels by undermining perceived control, which is one of the most fundamental resources in the JD–R theory. Violence and harassment emerged as recurrent stressors, often normalized within the sector, and inadequately addressed by employers. While previous reviews have described these risks, our synthesis demonstrates how they intersect with other organizational gaps, such as weak reporting mechanisms and poor managerial responsiveness, to amplify turnover intentions and reduce commitment, thereby damaging the safety of both HCWs and care receivers. These findings highlight the urgent need for training that addresses specific hazards, protective equipment, and systematic debriefing protocols that recognize the psychological and physical toll of unsafe environments.

The evidence also shows that HCWs heavily rely on personal strengths and self-initiated strategies, such as setting boundaries, regulating emotions, and taking proactive safety measures, to manage demands. From a JD–R perspective, these behaviors reflect resource-building, while W–HR clarifies how they support work–home enrichment. However, our synthesis highlights the limitations of this approach. When structural and organizational support is absent, individual coping mechanisms cannot compensate for chronic precarity, unsafe environments, or systemic devaluation. Reliance on self-sufficiency explains why resilience often appears fragile and why attrition persists despite strong personal commitment and intrinsic motivation.

### Devaluation of relational care

Perhaps the most distinctive contribution of this review is to show how efficiency-driven reforms may systematically devalue relational care. This pattern did not rest on a single isolated finding but on convergent evidence from several themes in the synthesis. More specifically, organizational change was associated with efficiency-oriented restructuring and the explicit prioritization of measurable, technical tasks over emotional and interpersonal aspects of care; this was reinforced by planning and scheduling practices that reduced the time available for meaningful interaction with clients. At the same time, the review showed that supportive, trust-based client–carer relationships were closely linked to emotional well-being, job satisfaction, motivation, and care quality, while time pressure and instrumental workload hindered opportunities to build and sustain such relationships. In JD–R terms, this dynamic represents a dual loss: it increases demands and removes a resource that could otherwise mitigate strain. From a W–HR perspective, the lack of time and recognition for relational labor also undermines recovery, as workers cannot draw on the emotional fulfilment and sense of meaning that relationships typically provide. This argument is also consistent with broader LTC scholarship showing that direct care work remains widely devalued despite requiring substantial relational skills and emotional labor (Scales, 2021).

This echoes Glenn’s (2010) notion of care work as “simultaneously priceless and worthless”—morally exalted yet economically undervalued. By situating this mechanism within an occupational health framework, our review shows how structural disregard for the relational aspects of care accelerates burnout, staff turnover, and low occupational esteem. This is in line with recent evidence from migrant HCWs showing that supportive and reciprocal caregiving relationships are central to care quality and well-being, whereas poor working conditions and unequal power dynamics can undermine these relational processes (Etxeberria et al., 2026).

### Limitations

The methodology used in the research on this topic shapes this review. First, most quantitative studies are cross-sectional, which limits the ability to draw causal inferences and observe demand-resource dynamics over time, as longitudinal or controlled evaluations remain scarce. Second, measurement variability (i.e., heterogeneous instruments for similar constructs) hindered direct comparison and precluded meta-analysis. Third, reporting gaps are common. Race/ethnicity, migration status, employment arrangements, and experiences of discrimination were not consistently documented, which complicates subgroup interpretation and likely underestimates inequities. Fourth, evidence on discrimination (e.g., racism, xenophobia, and sexism) was sparse and inconsistent, often confined to qualitative reports with limited quantification. Social desirability and fear of repercussions may further discourage reporting. Fifth, most participants were from North America and Northern/Western European countries, which restricts the generalizability of the findings to other cultural or geographic contexts. This review does not represent all HBC models equally. The findings are most applicable to paid HCWs caring for adult and older-adult populations in HBC settings and may be less applicable to pediatric HBC or short-term post-acute home health models, where care trajectories, worker roles, and organizational conditions may differ. Also, although qualitative and mixed-methods studies were generally rigorous, it is important to be aware that sensitive outcomes (e.g., violence or discrimination) may be underreported. At the same time, only 55.7% of the studies were theory-informed, and very few of these used gendered or intersectional frameworks. Consequently, mechanisms relating to gender, migration, and race may be under-specified in the primary evidence. Finally, the umbrella term “HCWs” encompasses diverse roles. While our model specifies moderators such as occupation, employment arrangement, and migration, future syntheses should stratify analyses and apply intersectional designs and measures to identify occupation- and group-specific levers.

### Implications

Our review identified several key areas for intervention. Relational care must be recognized and strengthened, for example, by allocating time for relationship-building and by making such work explicit in schedules, supervision, and appraisals. Measures like these are needed to counteract efficiency reforms that reduce care to instrumental tasks, thereby eroding a key motivational resource. Organizational buffers should be put in place to prevent routine demands from escalating into strain. This includes supervisory responsiveness, reliable pre-visit information, smooth service coordination, and reduced duplication in digital documentation. Workers must also be equipped for safe and competent practice by being given guaranteed access to appropriate equipment, specific training, and structured opportunities to debrief after emotionally challenging situations. Job limits must be supported with clear task definitions for clients and families, scheduling that avoids spillovers, and safeguards to ensure that flexibility does not translate into unpaid availability. Interventions should be tailored to employment arrangements: strengthening supervision, coordination, and safety climate in agency-hired contexts, and enforcing minimum protections and providing resources for guidance, training, and debriefing in direct-hire contexts. Finally, in line with the W–HR framework, predictable schedules and sufficient time off are important in enabling recovery. It is also necessary to recognize that time off is integral to employees’ well-being and ensuring the long-term quality of care.

## Conclusion

This MMSR integrates the JD–R and W–HR perspectives. It explains how multilevel conditions shape HCWs’ well-being. These conditions include the macro, organizational, job, relational, and personal levels. In short, evidence shows that high quantitative and emotional demands coupled with limited organizational resources contribute to strain, whereas supervision, coordination, appropriate equipment, training, and supportive relationships mitigate adverse well-being effects and sustain motivation. A key contribution of this review is drawing attention to the devaluation of relational care. Efficiency-driven changes reduce care work to instrumental tasks, increasing pressures while reducing time for meaningful connections, which represent a core motivational resource in the JD–R model and a recovery asset in the W–HR model. Most quantitative studies use cross-sectional designs. Qualitative and mixed-methods studies are generally rigorous and align with quantitative findings, meaning that conclusions are based on the weight of evidence rather than causality. In practice, improving well-being requires multilevel action, such as recognizing and facilitating relational care, ensuring supervisory responsiveness and coordination, providing appropriate equipment and role-relevant training, and reducing precarity through enforceable protections, especially in direct-hire contexts. Future research should prioritize longitudinal and intervention studies as well as consistent reporting of employment and migration status. It should also focus more on the gendered and intersectional dynamics of HBC to better understand the mechanisms and targets for change.

## Supplementary material

Supplementary material is available at *The Gerontologist* online.

## Funding

This work was supported by Horizon Europe “Care4Care: we care for those who care” project (grant number 101094603).

## Conflicts of interest

None declared.

## Supplementary Material

gnag131_Supplementary_Data

## Data Availability

This study was preregistered on PROSPERO (CRD42023492438). All the data extracted for this review synthesis is available from the authors upon request. We adhered to PRISMA guidelines, and the PRISMA checklist is included in the supplementary materials.
