# Supplementary material for: Antecedents of well-being of home care workers: a mixed-methods systematic review
Source: Gerontologist. 2026 Jun 20;66(8):gnag131. doi: 10.1093/geront/gnag131 (PMC13350945; doi:10.1093/geront/gnag131)
Supplement: gnag131_Supplementary_Data [file gnag131_supplementary_data.pdf]

Supplementary Material

Title: Antecedents of Well-Being of Home Care Workers: A Mixed-Methods Systematic Review

Authors: KURADCHIK-PEKARSKAYA, Veronika; MARTINEZ-CORTS, Ines; GAGO-VALLE, Celia; MEDINA, Francisco Jose

Table of contents

Supplementary Material – File 1: PRISMA Checklist 2

Supplementary Material – File 2: Complete search syntax for each database 5

Supplementary material – File 3: Characteristics of included studies 9

Supplementary material – File 4: MMAT tool 2018 version for selected studies 24

Supplementary Material – File 1: PRISMA Checklist

Section and Topic	Item #	Checklist item	Location where item is reported
**TITLE**			
Title	1	Identify the report as a systematic review.	Title Page
**ABSTRACT**			
Abstract	2	See the PRISMA 2020 for Abstracts checklist.	Abstract section
**INTRODUCTION**			
Rationale	3	Describe the rationale for the review in the context of existing knowledge.	Background section
Objectives	4	Provide an explicit statement of the objective(s) or question(s) the review addresses.	Objectives section
**METHODS**			
Eligibility criteria	5	Specify the inclusion and exclusion criteria for the review and how studies were grouped for the syntheses.	Inclusion and Exclusion Criteria section
Information sources	6	Specify all databases, registers, websites, organisations, reference lists and other sources searched or consulted to identify studies. Specify the date when each source was last searched or consulted.	Search Methods section
Search strategy	7	Present the full search strategies for all databases, registers and websites, including any filters and limits used.	Search Methods section
Selection process	8	Specify the methods used to decide whether a study met the inclusion criteria of the review, including how many reviewers screened each record and each report retrieved, whether they worked independently, and if applicable, details of automation tools used in the process.	Search Outcome section
Data collection process	9	Specify the methods used to collect data from reports, including how many reviewers collected data from each report, whether they worked independently, any processes for obtaining or confirming data from study investigators, and if applicable, details of automation tools used in the process.	Data Abstraction section
Data items	10a	List and define all outcomes for which data were sought. Specify whether all results that were compatible with each outcome domain in each study were sought (e.g. for all measures, time points, analyses), and if not, the methods used to decide which results to collect.	Data Abstraction section
	10b	List and define all other variables for which data were sought (e.g. participant and intervention characteristics, funding sources). Describe any assumptions made about any missing or unclear information.	Data Abstraction section
Study risk of bias assessment	11	Specify the methods used to assess risk of bias in the included studies, including details of the tool(s) used, how many reviewers assessed each study and whether they worked independently, and if applicable, details of automation tools used in the process.	Quality Appraisal section
Effect measures	12	Specify for each outcome the effect measure(s) (e.g. risk ratio, mean difference) used in the synthesis or presentation of results.	N/A
Synthesis methods	13a	Describe the processes used to decide which studies were eligible for each synthesis (e.g. tabulating the study intervention characteristics and comparing against the planned groups for each synthesis (item #5)).	Synthesis section
	13b	Describe any methods required to prepare the data for presentation or synthesis, such as handling of missing summary statistics, or	Synthesis section

Section and Topic	Item #	Checklist item	Location where item is reported
		data conversions.	
	13c	Describe any methods used to tabulate or visually display results of individual studies and syntheses.	Results of Synthesis section
	13d	Describe any methods used to synthesize results and provide a rationale for the choice(s). If meta-analysis was performed, describe the model(s), method(s) to identify the presence and extent of statistical heterogeneity, and software package(s) used.	N/A
	13e	Describe any methods used to explore possible causes of heterogeneity among study results (e.g. subgroup analysis, meta-regression).	N/A
	13f	Describe any sensitivity analyses conducted to assess robustness of the synthesized results.	N/A
Reporting bias assessment	14	Describe any methods used to assess risk of bias due to missing results in a synthesis (arising from reporting biases).	N/A
Certainty assessment	15	Describe any methods used to assess certainty (or confidence) in the body of evidence for an outcome.	N/A
**RESULTS**			
Study selection	16a	Describe the results of the search and selection process, from the number of records identified in the search to the number of studies included in the review, ideally using a flow diagram.	Study Selection section
	16b	Cite studies that might appear to meet the inclusion criteria, but which were excluded, and explain why they were excluded.	Study Selection section
Study characteristics	17	Cite each included study and present its characteristics.	Study Characteristics section
Risk of bias in studies	18	Present assessments of risk of bias for each included study.	Risk of Bias section
Results of individual studies	19	For all outcomes, present, for each study: (a) summary statistics for each group (where appropriate) and (b) an effect estimate and its precision (e.g. confidence/credible interval), ideally using structured tables or plots.	Study Characteristics section
Results of syntheses	20a	For each synthesis, briefly summarise the characteristics and risk of bias among contributing studies.	Results of Syntheses section
	20b	Present results of all statistical syntheses conducted. If meta-analysis was done, present for each the summary estimate and its precision (e.g. confidence/credible interval) and measures of statistical heterogeneity. If comparing groups, describe the direction of the effect.	N/A
	20c	Present results of all investigations of possible causes of heterogeneity among study results.	N/A
	20d	Present results of all sensitivity analyses conducted to assess the robustness of the synthesized results.	N/A
Reporting biases	21	Present assessments of risk of bias due to missing results (arising from reporting biases) for each synthesis assessed.	N/A
Certainty of evidence	22	Present assessments of certainty (or confidence) in the body of evidence for each outcome assessed.	N/A
**DISCUSSION**			
Discussion	23a	Provide a general interpretation of the results in the context of other evidence.	Discussion section
	23b	Discuss any limitations of the evidence included in the review.	Limitations section
	23c	Discuss any limitations of the review processes used.	Limitations section

Section and Topic	Ite m #	Checklist item	Location where item is reported
	23d	Discuss implications of the results for practice, policy, and future research.	Practical Implications and Conclusion sections
**OTHER INFORMATION**			
Registration and protocol	24a	Provide registration information for the review, including register name and registration number, or state that the review was not registered.	Design section
	24b	Indicate where the review protocol can be accessed, or state that a protocol was not prepared.	Design section
	24c	Describe and explain any amendments to information provided at registration or in the protocol.	N/A
Support	25	Describe sources of financial or non-financial support for the review, and the role of the funders or sponsors in the review.	Funding Statement section
Competing interests	26	Declare any competing interests of review authors.	Conflict of Interest section
Availability of data, code and other materials	27	Report which of the following are publicly available and where they can be found: template data collection forms; data extracted from included studies; data used for all analyses; analytic code; any other materials used in the review.	Acknowledgements section

Supplementary Material – File 2: Complete search syntax for each database

Database: ProQuest APA PsycInfo

- Date of the search: April 8th, 2026
- Records retrieved: **611**
- Exact strategy used in “Advanced Search”:
    1. Abstract("home caregiver*" OR "domestic service worker*" OR "professional carer*" OR "homebound care" OR "health care service*" OR "basic care worker*" OR "home care" OR "paid caregiver*" OR "remunerated caregiver*" OR "professional caregiver*" OR "formal caregiver*" OR "personal care aide*" OR "professional home care provider*" OR "domestic care worker*" OR "direct care worker*" OR "paid home care staff" OR "home health aide*" OR "home support worker*" OR "home health care" OR "home nurs*" OR "home health nurs*" OR "visiting nurs*" OR "community nurs*")
    2. Abstract("working conditions" OR "emotional adjustment" OR "emotional regulation" OR "self-care" OR "self-efficacy" OR "empowerment" OR "self-management" OR "coping behavior" OR "psychosocial factors" OR "motivation")
    3. Abstract("well-being" OR "health" OR "physical health" OR "mental health" OR "stress" OR "burnout" OR "anxiety" OR "depression" OR "satisfaction" OR "life" satisfaction " OR " quality of life "")
    4. Boolean operators: AND for the three rows above
    5. Filters/limits applied:
        - Language: English or Spanish
        - Publication date: 2000 – current (2026)
        - Peer reviewed: Yes

Database: Clarivate Web of Science

- Date of the search: April 8th, 2026
- Records retrieved: **1,920**
- Exact strategy used in "Advanced Search":
    1. **Row 1 – Sample**: abstract("home caregiver*" OR "domestic service worker*" OR "professional carer*" OR "homebound care" OR "health care service*" OR "basic care worker*" OR "home care" OR "paid caregiver*" OR "remunerated caregiver*" OR "professional caregiver*" OR "formal caregiver*" OR "personal care aide*" OR "professional home care provider*" OR "domestic care worker*" OR "direct care worker*" OR "paid home care staff" OR "home health aide*" OR "home support worker*" OR "home health care" OR "home nurs*" OR "home health nurs*" OR "visiting nurs*" OR "community nurs*")
    2. **Row 2 – Phenomenon of interest**: abstract("working conditions" OR "emotional adjustment" OR "emotional regulation" OR "self-care" OR "self-efficacy" OR "empowerment" OR "self-management" OR "coping behavior" OR "psychosocial factors" OR "motivation")
    3. **Row 3 – Evaluation/well-being outcomes**: abstract("well-being" OR "health" OR "physical health" OR "mental health" OR "stress" OR "burnout" OR "anxiety" OR "depression" OR "satisfaction" OR "life" satisfaction " OR " quality of life "")
    4. Boolean operators: AND for the three rows above
    5. Filters/limits applied:
        - Language: English or Spanish
        - Publication date: 2000-01-01 – current (2026-12-31)
        - Document Types: Article
        - Database: Web of Science Core Collection

Database: Ovid MEDLINE

- Date of the search: April 8th, 2026
- Records retrieved: **1,723**
- Exact strategy used in “Advanced Search”:
    1. ("home caregiver*" OR "domestic service worker*" OR "professional carer*" OR "homebound care" OR "health care service*" OR "basic care worker*" OR "home care" OR "paid caregiver*" OR "remunerated caregiver*" OR "professional caregiver*" OR "formal caregiver*" OR "personal care aide*" OR "professional home care provider*" OR "domestic care worker*" OR "direct care worker*" OR "paid home care staff" OR "home health aide*" OR "home support worker*" OR "home health care" OR "home nurs*" OR "home health nurs*" OR "visiting nurs*" OR "community nurs*").ab.
    2. ("working conditions" OR "emotional adjustment" OR "emotional regulation" OR "self-care" OR "self-efficacy" OR "empowerment" OR "self-management" OR "coping behavior" OR "psychosocial factors" OR "motivation").ab.
    3. (“well-being" OR "health" OR "physical health" OR "mental health" OR "stress" OR "burnout" OR "anxiety" OR "depression" OR "satisfaction" OR "life" satisfaction " OR " quality of life").ab.
    4. 1 AND 2 AND 3
    5. Limit 4 to English Language
    6. Limit 4 to Spanish
    7. 5 OR 6
    8. limit 7 to yr="2000 -Current"
    9. limit 8 to "remove preprint records"
    10. (review or meta-analysis).pt.
    11. 9 NOT 10

Database: EBSCOhost CINAHL Complete

- Date of the search: April 8th, 2026
- Records retrieved: **1,127**
- Exact strategy used in “Advanced Search”:
    1. Abstract("home caregiver*" OR "domestic service worker*" OR "professional carer*" OR "homebound care" OR "health care service*" OR "basic care worker*" OR "home care" OR "paid caregiver*" OR "remunerated caregiver*" OR "professional caregiver*" OR "formal caregiver*" OR "personal care aide*" OR "professional home care provider*" OR "domestic care worker*" OR "direct care worker*" OR "paid home care staff" OR "home health aide*" OR "home support worker*" OR "home health care" OR "home nurs*" OR "home health nurs*" OR "visiting nurs*" OR "community nurs*")
    2. Abstract ("working conditions" OR "emotional adjustment" OR "emotional regulation" OR "self-care" OR "self-efficacy" OR "empowerment" OR "self-management" OR "coping behavior" OR "psychosocial factors" OR "motivation")
    3. Abstract (“well-being" OR "health" OR "physical health" OR "mental health" OR "stress" OR "burnout" OR "anxiety" OR "depression" OR "satisfaction" OR "life" satisfaction " OR " quality of life")
    4. limit 7 to yr="2000 – Current (04/2026)"
    5. Limit languages: English or Spanish
    6. Type of publication: journal article, peer-reviewed records
    7. Source: academic journals

Supplementary material – File 3: Characteristics of included studies

Table 1 of supporting information

Characteristics of included studies

Nº	Citation and country	Study design	Aim	Participants	Key codes
1	Agbonifo et al. (2017) USA	QUAN	To identify health and safety risks and associated health problems for HCWs during patient visits and explore prevention strategies to reduce injuries, illnesses, and fatalities.	31 home healthcare nurses, 23 home health aides, and 4 physical and occupational therapists. Mostly women, aged 40 or over, mostly white, with an average of 10.6 years in the profession and 5.4 years with their current employer.	***Demands***: physical demands, injury hazards; biological, chemical and environmental exposures<br>***Outcomes***: musculoskeletal well-being; respiratory, cardiovascular, and dermatological system diseases
2	Andersen & Westgaard (2015) Norway	MIX	To examine potential organisational differences in the assessment of factors affecting the working conditions of HCWs.	80 HCWs completed the survey. In addition, 33 participants took part in semi-structured interviews, representing stakeholders at three organisational levels: HCWs, unit managers and municipal representatives.	***Demands***: time pressure, workload, cut-backs, precariousness<br>***Resources***: social support, employer support<br>***Outcomes***: quality of care, frustration, sick leave
3	Aronsson et al. (2014) Sweden	QUAN	To identify factors associated with stress accumulation and poor recovery, and by exploring the relationship between recovery and health.	67 HCWs; 74 social workers; 52 preschool workers (only HCWs were of interest for this review). Predominantly female, married, 46-55 years old, 10 years of tenure, and had permanent and part-time contracts.	***Demands***: workload (pace and intensity), time pressure<br>***Resources***: control, social support, satisfaction with one's work and access to resources, role clarity<br>***Outcomes***: physical and mental recovery, emotional ill-being, sick leave, general health
4	Ayalon (2009) Israel	MIX	To assess the working conditions and exposure to workplace violence of Filipino HCWs in Israel, and related clinical outcomes such as burnout.	245 Filipino HCWs, mostly women, average age 36. Most had some college or vocational training, were unmarried and had lived in Israel for 1-5 years. 29 Filipino HCWs participated in the interviews.	***Demands***: violence, precariousness, tasks beyond duties, legal awareness<br>***Resources***: social support outside of work, occupational esteem<br>***Outcomes***: burnout, injury
5	Ayalon (2012) Israel	QUAN	To assess suicidal ideation and attempts (SIA) and depressive symptoms in a sample of Filipino migrant HCWs in Israel.	178 Filipino HCWs. Majority female, age 37.7 years, married, live-in HCWs, average 8.2 years of education and average 4.4 years of residence in Israel.	***Demands***: abuse<br>***Resources***: social support<br>***Outcomes***: depressive symptoms and SIA

Table 1 of supporting information

Characteristics of included studies

Nº	Citation and country	Study design	Aim	Participants	Key codes
6	Bagnasco et al. (2024) Italy	QUAN	To describe HBC nurses' characteristics, working conditions, missed care, and patients' care experience.	2549 HBC nurses (79.5% women; mean age 46.6); employed in the public sector; most had a regional diploma or bachelor's degree; 27.6% had postgraduate training in home or community nursing.	***Demands***: workload pressures (high number of visits and short duration), staff shortage ***Outcomes***: quality of care (missed care), job satisfaction, turnover intentions
7	Bakker et al. (2003) The Netherlands	QUAN	To test and extend the Job Demands-Resources (JD-R) model of burnout by examining how job demands and resources interact to influence burnout dimensions among HCWs.	3,092 Home care assistants/aides who support clients with physical or mental health conditions who need help with activities of daily living (instrumental, emotional and informational support); mostly female, average age 42, with 10 years' experience.	***Demands***: workload, physical, emotional, harassment, schedule uncertainty ***Resources***: skill discretion, decision authority, social support, feedback, financial rewards, possibilities for development ***Outcomes***: burnout (exhaustion, cynicism, professional efficacy)
8	Barken et al. (2015) Canada	QUAL	To examine how task-shifting affects the skills of HCWs.	20 HCWs, 9 HCW supervisors, 9 therapists (5 occupational therapists and 4 physiotherapists) and 8 nurses. All women.	***Demands***: task shifting, precariousness, time pressure ***Resources***: cognitive abilities, occupational esteem, control autonomy, training, client-carer relationship, skill discretion, meaning ***Outcomes***: quality of care
9	Brulin et al. (2000) Sweden	QUAL	To investigate the work environment factors that HCWs perceive as challenging and problematic.	8 female HCWs with musculoskeletal complaints of the neck, shoulders, upper back and/or lower back in their 1995 survey.	***Demands***: safety hazards, cut-back, workload, time pressure, uncertainty, precariousness, physical demands ***Resources***: control, social support, autonomy, variety, self-organising teamwork, training, supervisor support ***Outcomes***: stress, MSDs, quality of care, job satisfaction, fear of injuring clients, frustration
10	Butler (2018) USA	MIX	To explore the factors influencing turnover among HC aides and to examine how both those who stayed and those who left experienced their work.	The study surveyed 261 HCWs and conducted interviews with 252 participants. The sample was predominantly female, mostly white, aged 46 years on average, and reported very low household incomes.	***Demands***: safety hazards, physical demands, violence, death, difficult clients ***Resources***: empowerment, training, agency responsiveness, autonomy, personal safety behaviour ***Outcomes***: job satisfaction, burnout, turnover, occupational injury, sick leave

Table 1 of supporting information

Characteristics of included studies

Nº	Citation and country	Study design	Aim	Participants	Key codes
11	Butler et al. (2010) USA	MIX	To investigate factors associated with job termination among personal assistants in HBC.	261 personal support workers completed an initial survey. 70 of these took part in a follow-up survey and interviews. The sample was predominantly female, mostly white, with an average age in the mid-40s. Participants reported very low household incomes, and many had no health insurance.	***Demands***: precariousness, conflict, difficult clients ***Resources***: employer support, client-carer relationship ***Outcomes***: turnover intention, burnout, job satisfaction, health, work-home spillover
12	Butler et al. (2012) USA	MIX	To explore the work experiences, including benefits and challenges, and to identify predictors of job termination among personal assistant workers in HBC.	The primary sample included 261 personal support workers, mostly women around 46 years old, predominantly white, with low household incomes and limited health insurance coverage. 88 of those who left their jobs participated in follow-up interviews.	***Demands***: workload, precariousness, death, violence ***Resources***: psychological empowerment, client-carer relationship, meaning, autonomy, employer support ***Outcomes***: turnover, burnout, job satisfaction, health
13	Cacciapuoti et al. (2025) Italy	QUAL	To explore the experiences of HCWs assisting clients with chronic diseases focusing on feelings and working conditions.	17 migrant HCWs (all women; mean age 54; mostly from Romania, Moldova, Ukraine, Philippines, and Sri Lanka). Most were live-ins; 15 had no formal training. Type of employment not specified.	***Demands***: job insecurity ***Resources***: client-carer relationship, non-work social support ***Outcomes***: emotional well-being
14	Chang et al. (2025) South Korea	QUAN	To examine the influence of structural empowerment on psychological empowerment through thriving at work and caregiver reciprocity.	192 HCWs, mostly middle-aged women, with college (or higher) education and at least with a high school diploma, all with national certification, trained to provide personal care, ADL support, and basic health monitoring in clients' homes.	***Resources***: opportunities for development, access to information, support from peers/supervisors, employer support, client-carer relationship (caregiver reciprocity) ***Outcomes***: thriving at work
15	Chowdhury & Gutman (2012) Canada	MIX	To deepen understanding of Live-in Caregiver Programme (LCP) care workers and their older adult clients, exploring career trajectories and changes in job and life satisfaction over time.	14 Filipino live-in HCWs, aged 15-64, most with university degrees (mainly in nursing and education) and half married. All migrated to Canada between 2004 and 2008. Most had gained skills for the Live-in Caregiver Programme through training and work in long-term care; four had completed caregiver diploma courses in the Philippines.	***Demands***: job complexity, precariousness, time pressure, job insecurity, violence, difficult clients ***Resources***: meaning, benefits, support, possibilities for development ***Outcomes***: life and job satisfaction, turnover intentions, emotional well-being, sleep problems, quality of care

Table 1 of supporting information

Characteristics of included studies

Nº	Citation and country	Study design	Aim	Participants	Key codes
16	Cindrić & Malnar (2025) Croatia	QUAN	To determine how organisational support structures influence job satisfaction in physically demanding working environments by comparing urban and rural settings.	517 HCWs, mostly nurses and nursing assistants, along with some physiotherapists and social care workers. All were exposed to physical effort, moving/lifting heavy loads and travelling between care receivers' homes.	***Demands***: physical demands ***Resources***: employer support, supportive leadership, autonomy, training, access to information, planning ***Outcomes***: job satisfaction
17	Delp & Muntaner (2011) USA	MIX	To analyse the work situation of Californian HCWs, drawing up a profile of the workforce and examining work stressors, support and control, with a focus on differences by gender, race and ethnicity, and the role of policy - particularly unionisation - in shaping their experiences.	1,614 HCWs. Mostly female, average age 52, married, migrant and caring for a single client.	***Demands***: physical, emotional, schedule uncertainty, workload pressures, hazardous work environment ***Resources***: control, support, benefits ***Outcomes***: mental and physical health outcomes, job satisfaction
18	Denton et al. (2002) Canada	MIX	To investigate how working in clients' homes affects the mental health and well-being of visiting HCWs, focusing on stress and intrinsic job satisfaction.	674 visiting staff (nurses, therapists and home support workers) from three not-for-profit HC agencies, mostly women. Nurses and therapists tended to be older and better educated than HCWs. 99 HCWs took part in focus groups.	***Demands***: workload, time pressure, precariousness, emotional demands, safety hazards, violence, physical demands ***Resources***: variety, employer support and benefits, predictability, social support, control, client-carer relationship ***Outcomes***: job stress, intrinsic job satisfaction, health, injury
19	Denton et al. (2015) Canada	QUAL	To examine HCWs' perspectives on how task shifting affects the quality of care provided to older adults.	46 home healthcare workers (aides, supervisors, nurses and therapists). All were women, mostly in their late 40s. About half of the aides and nurses and a third of the therapists worked part-time, while all the supervisors worked full-time.	***Demands***: task shifting, schedule demands, workload ***Resources***: flexibility, client-carer relationship, training, supervisor support ***Outcomes***: quality of care
20	Fernández-Carrasco et al. (2022) Spain	QUAN	To evaluate the quality of life and anxiety levels experienced by Latin American immigrants in Spain who care for older adults.	426 Latin American immigrant personal care workers in Spain, mostly women with a mean age of 36 and mostly without formal education in care services; the sample included both live-in and non-live-in workers.	***Demands***: precarious employment, workload pressures, physical demands, emotional demands (discrimination) ***Outcomes***: quality of life, anxiety

Table 1 of supporting information

Characteristics of included studies

Nº	Citation and country	Study design	Aim	Participants	Key codes
21	Fleming & Taylor (2006) UK	MIX	To explore the factors influencing the retention of HCWs within a health and social care trust in Northern Ireland.	45 HCWs completed questionnaires and 12 took part in focus groups to gain deeper insights. All participants were women, aged 47.9 years on average, with varying levels of experience and mostly without formal qualifications.	***Demands***: workload, precariousness, tasks beyond duties ***Resources***: supervisor support, appreciation, occupational esteem, employer support, self-efficacy, meaning, benefits, onboarding ***Outcomes***: turnover intention, job satisfaction, work-home spillover, quality of care
22	Franzosa et al. (2019) USA	QUAL	To examine home health aides' perceptions of the emotional effects of their work, their coping mechanisms and the support they desire.	27 home health aides, 92,6% women, 78% were foreign-born, the sample was highly experienced, and most were currently caring for 1-3 long-term clients.	***Demands***: difficult clients, workload pressures, precarious employment ***Resources***: client-carer relationship, job meaning, supportive leadership, occupational esteem, co-worker support, non-work social support ***Outcomes***: emotional ill-being, work-home spillover
23	Ghoroubi et al. (2023) France	QUAN	To assess potential work-related exposure to SARS-CoV-2 across occupational groups in France before the first lockdown.	Data from two national surveys (CT, n = 23,231; SUMER, n = 26,297) were used. The study provides a weighted estimate of 397,736 workers in the "home carers and domestic helpers" group (391,011 women, 13,800 men).	***Demands***: safety hazards (infectious agents)
24	Green & Ayalon (2018) Israel	QUAN	To examine the working conditions and prevalence of abuse and exploitation among live-in migrant and live-out HCWs.	338 migrant live-in HCWs and 185 local live-out HCWs. In both groups, the majority were women, married and had completed secondary education. The average age was 38.9 years for live-in workers and 53.1 years for live-out workers.	***Demands***: violation of workers' rights, workplace violence, emotional
25	Gusoff et al. (2025) USA	QUAL	To investigate perceived factors contributing to job quality and retention among workers at HBC cooperatives.	23 HCWs and 9 staff members (total = 32). Mostly women, ethnically diverse (25% African American, 13% Latin American, 53% White); ages ranged from 20 to 70+. All employed by cooperatives across five US regions, primarily as worker-owners.	***Resources***: influence and autonomy, type of employment (cooperatives), benefits, co-worker support, recognition ***Outcomes***: retention

Table 1 of supporting information

Characteristics of included studies

Nº	Citation and country	Study design	Aim	Participants	Key codes
26	Hanson et al. (2015) USA	QUAN	To assess the prevalence of workplace violence among home care workers and explore its associations with their stress, burnout, depression and sleep outcomes.	1,214 home care workers, predominantly women, average age 47.3 years, majority white, most with a high school diploma; average tenure 7.9 years, working about 33.5 hours per week.	***Demands***: workplace violence<br>***Resources***: confidence to prevent and respond to violence<br>***Outcomes***: fear of violence, burnout, stress, depression, sleep problems
27	Hittle et al. (2016) USA	QUAN	To determine occupational exposures for HCWs (home health-care nurses and aides).	31 home health nurses (HHNs) and 23 home health aides (HHAs), all women aged 40 or older, mostly white. HHNs had an average of 8.5 years in HBC and 4.0 years with their current employer; HHAs had an average of 13.9 years in total and 7.7 years with their current employer.	***Demands***: physical demands, injury hazards; biological, chemical and environmental exposures
28	Hsu & Chen (2025) Taiwan	QUAN	To study the mutual relationships and satisfaction among care receivers, family carers, and live-in migrant care workers.	156 live-in migrant care workers (care receiver and family carers were excluded from the review). All women; mean age 34.5; 74% from Indonesia, 20% Philippines, 6% Vietnam. Most had around 4 years of experience and cared for people with physical or cognitive impairments. All directly employed by families through Taiwan's LTC system.	***Demands***: working conditions (private room)<br>***Resources***: enough rest time, job meaning (positive feeling from caregiving), social support from employer<br>***Outcomes***: job satisfaction
29	Janssen & Abbott (2023) USA	QUAL	To describe the facilitators and resources that support the emotional health of HCWs.	17 female HCWs, with 1.5 to 22 years in their current agency (average 8.3 years) and 2 to 33 years in the care profession overall (average 10.5 years).	***Resources***: self-regulation, self-care, social support outside of work, benefits, satisfaction with wages, training, development<br>***Outcomes***: emotional health, work-life spillover
30	Jepsen et al. (2025) Australia	QUAL	To identify the range of WHS hazards and harms that HCWs face, as well as the risk factors that mitigate or exacerbate them.	Thirty-five HCWs, mostly women, with both local (n=18) and migrant (n=17) workers. Thy were employed across standard providers, digital platforms, labour hire, and independent arrangements.	***Demands***: exposure to violence, time pressure, physical demands, safety hazards<br>***Resources***: training, employer support, client-carer relationship<br>***Outcomes***: physical ill-being, psychological ill-being

Table 1 of supporting information

Characteristics of included studies

Nº	Citation and country	Study design	Aim	Participants	Key codes
31	Karlsson et al. (2020) USA	MIX	To examine HC aides' experiences of clients requesting tasks outside their job description and the association of this phenomenon with occupational safety and health outcomes.	954 HC aides, mostly women with a mean age of 48 years. Qualitative data came from nine focus groups (70 aides) and seven interviews with industry and worker representatives, also predominantly female.	***Demands***: tasks beyond duties, violence, safety hazards, physical demands, conflict, precariousness<br>***Resources***: benefits, meaning, client-carer relationship, social support, occupational esteem<br>***Outcomes***: job satisfaction, injury/pain, turnover
32	Kelly et al. (2024) Canada	QUAL	To examine the factors influencing HCWs' decisions to work directly for clients or via agencies.	20 HCWs (16 women, 4 men; majority had >10 years of experience; 8 migrants; broad range of education levels including foreign-trained nurses). 15 worked in directly-funded care (hired by clients), 5 via agencies.	***Demands***: emotional demands, racism, insecurity<br>***Resources***: benefits, autonomy, flexibility, supervisor support, confidence and experience, client-carer relationship<br>***Outcomes***: attraction to the job
33	Koivula et al. (2016) Finland	QUAL	To explore HCWs' experiences of alcohol-related situations, their responses and their perceptions of how they manage them.	10 HCWs (registered nurses, public health nurses and practical nurses) caring for older people who use alcohol in Finland.	***Demands***: drunk clients, tasks beyond duties, conflict<br>***Resources***: employer support, training<br>***Outcomes***: worry
34	Kriegsmann-Rabe et al. (2023) Germany	QUAL	To explore stressors and resilience factors affecting the well-being of migrant live-in migrant HCWs living in Germany.	16 migrant home care workers (all Polish and all but one female) with an average age of 55 (range: 36-68). Mean of 12.6 years of experience as a live-in in Germany.	***Demands***: permanent availability, overwork, tasks beyond duties, death, violence, discrimination, difficult clients, physical demands, precariousness<br>***Resources***: self-determination, skill, intrinsic job motivation, social support outside of work, client-carer relationship, free time<br>***Outcomes***: well-being, psychological resilience, turnover, work-life spillover
35	Kusmaul et al. (2020) USA	QUAL	To explore how HCWs experience and define empowerment in their roles, and to identify ways to enhance it.	12 female HCWs (certified nursing assistants and personal care workers), aged 20 to 64 years (mean age 42.5 years). Their home care experience ranged from 2 weeks to 40 years (mean 12.9 years), and 11 had health insurance.	***Demands***: sexual violence, difficult clients, precarious employment<br>***Resources***: structural empowerment, psychological empowerment, occupational esteem, autonomy, training<br>***Outcomes***: job satisfaction, health, turnover, client satisfaction

Table 1 of supporting information

Characteristics of included studies

Nº	Citation and country	Study design	Aim	Participants	Key codes
36	Larsson et al. (2012) Sweden	QUAN	To identify factors facilitating work ability and self-efficacy of HCWs (nurse aides and nursing assistants).	58 care aides and 79 assistant nurses. The majority were female, and the average age was 45.	***Demands***: physical and psychosocial (pace, effort, time pressure, role conflict) ***Resources***: personal safety, self-efficacy, safety climate ***Outcomes***: musculoskeletal well-being, work ability
37	Larsson et al. (2013) Sweden	QUAN	To explore HCWs' views on health, workplace risks, working conditions and how risks are managed within their organisation.	133 home care workers (aides and nursing assistants), mostly women, average age 45, employed full-time on permanent contracts, with an average of 12.4 years' experience in home care and an average unit size of 26.1 staff.	***Demands***: workload, time pressure, complexity ***Resources***: social support, decision-making authority, safety climate ***Outcomes***: physical and emotional well-being, work ability, musculoskeletal, job strain
38	Lee & Jang (2016) USA	QUAN	To explore factors influencing HCWs' intention to leave, using the JD-R model to examine how physical injury and organisational support affect turnover intention.	150 home health workers. Predominantly female, non-white minorities, low income.	***Demands***: hazardous work environment ***Resources***: organisational support ***Outcomes***: injury, musculoskeletal disorders, turnover
39	Lee & Oh (2023) South Korea	QUAN	To identify the working conditions that influence the psychological outcomes of paid family and non-family HCWs.	298 paid family HCWs and 700 non-family HCWs. Majority female, in their 50s and 60s, without higher education, working part-time and for a single agency.	***Demands***: workload, overtime work, verbal abuse ***Outcomes***: stress, turnover, injury/illness
40	Liaset et al. (2024) Norway	QUAL	To explore HCWs' experiences of using different modes of transport for client visits, and their perceived health effects.	14 HCWs from one municipal HBC unit in Trondheim (5 assistant nurses, 5 nurses, 4 other health professionals). 79% women; mean age 37.4. All were publicly employed.	***Demands***: emotional demands, physical demands, safety hazards ***Resources***: rest ***Outcomes***: physical and mental well-being
41	Lindholm et al. (2021) Sweden	QUAN	To identify the links between occupational physical and psychosocial factors and sleep-related problems among HCWs.	665 HCWs. Predominantly female, mean age 44.3 years, completed secondary education, with experience in HBC of ≤5 years (42.6%), 6–9 years (15.0%), ≥10 years (40.2%).	***Demands***: qualitative, quantitative, physical, emotional, clients' diagnosis ***Resources***: leadership, job contentment ***Outcomes***: sleep and musculoskeletal

Table 1 of supporting information

Characteristics of included studies

Nº	Citation and country	Study design	Aim	Participants	Key codes
42	Lindquist et al. (2012) USA	QUAL	To explore what motivates someone to become paid carers, their perceptions of working conditions, and how their motivation may be related to negative health outcomes in older individuals.	98 non-family paid carers. The average age was 49.5 years and most were women. The majority were foreign-born and had no family or support networks in the US or locally.	***Demands***: physical demands, death, client-carer relationship<br>***Resources***: meaning<br>***Outcomes***: stress, depression
43	Lohne et al. (2024) Norway	QUAN	To examine whether patients' ADL self-care score predicts HCWs' standing time during visits.	14 HCWs (11 women, 3 men); occupations included 5 nurses, 4 assistant nurses, 2 occupational therapists, 2 welfare nurses, and 1 physiotherapist; mean age 37.3 years; all employed in the public sector.	***Demands***: physical demands (standing), clients' ADL needs
44	Love et al. (2017) USA	QUAL	To explore how HCWs perceive and manage their risks of musculoskeletal disorders (MSDs), with the aim of informing prevention strategies and workplace policies.	37 HCWs (home care workers and personal care workers) Predominantly female and African American with an average age of 43.	***Demands***: safety hazards, physical demands, emotional demands<br>***Resources***: cognitive abilities, client-carer relationship, social support, training<br>***Outcomes***: musculoskeletal, fear of injury, stress, STFs, quality of care
45	Markkanen et al. (2007) USA	QUAL	To qualitatively characterise the work experience and hazards of HCWs, with a focus on risk factors for bloodborne pathogen exposure.	17 nurses and 7 home health aides in non-supervisory positions with experience in handling sharps or working in homes at risk of bloodborne pathogen exposure.	***Demands***: safety hazards, physical demands, workload pressures, uncertainty, dealing with death<br>***Resources***: co-worker support, job meaning, flexibility
46	Markkanen et al. (2014) USA	QUAL	To examine the work context and safety hazards for HC aides, identify effective practices and policies to improve safety for both clients and aides, and examine the links between client and aide safety and risks from tasks beyond official duties.	99 home care aides (mostly female, white) with experience ranging from a few months to over 30 years.	***Demands***: physical and emotional, safety hazards, violence, discrimination, tasks beyond duties, conflict, precariousness, overwork<br>***Resources***: occupational esteem, social support client-carer relationship, autonomy<br>***Outcomes***: musculoskeletal health, turnover, job satisfaction, quality of care, STF, strain
47	Markkanen et al. (2017) USA	QUAL	To summarise the main occupational safety and health (OSH) risks in HBC, particularly those related to home infusion therapy.	Home health nurses and aides involved in infusion therapy (8 focus groups), plus 29 home care providers and 35 agency managers, industry directors and union representatives.	***Demands***: safety risks, difficult clients, distractions, use of technology, physical demands<br>***Resources***: access to lifting equipment<br>***Outcomes***: sharps injury, frustration, musculoskeletal health

Table 1 of supporting information

Characteristics of included studies

Nº	Citation and country	Study design	Aim	Participants	Key codes
48	Martínez-Buján & Moré (2024) Spain	QUAL	To examine the role of intermediary agencies and digital platforms in brokering HBC and their impact on migrant workers' working conditions.	10 migrant HCWs (live-in and love-out, direct (through broker) and agency-hired, mostly women, Latin American, aged 50+, no training), 6 managers, 4 representatives of HBC associations.	***Demands***: employment insecurity, workload pressures ***Resources***: trade unions, employer support, training
49	Martínez-de la Torre et al. (2025) Spain	QUAL	To explore and describe the experiences of home care workers caring for people with Alzheimer's disease, and to identify relevant personal factors.	15 home care assistants, all female, with an average age of 48 years, over 1 year of experience and specific training in home care in urban and rural settings. Their job tasks included providing basic care, accompaniment and cleaning household tasks.	***Demands***: clients' diagnosis, physical demands, precarious employment, emotional demands ***Resources***: occupational esteem, employer support, training, client-carer relationship, job meaning ***Outcomes***: pain and fatigue, stress, mental fatigue, job satisfaction
50	Minguela-Recover et al. (2022) Spain	MIX	This study aims to examine the well-being of HCWs in different service management models and to explore their emotional experiences related to their work and working conditions.	15 female HCWs, mean age 47.3 years, all with more than 10 years' experience in the Home Help Service. All had experienced the 2012 labour conflict and worked during the COVID-19 pandemic. All had a Home Care Professional Certificate; almost half had specialist training in social and health care.	***Demands***: complexity, death, workload pressure, tasks beyond duties, precariousness, physical demands, travel demands ***Resources***: meaning of the job, possibilities for development, employer support, flexibility, social support from families ***Outcomes***: Happiness, stress, work-life spillover, physical exhaustion
51	Mitchell et al. (2023) UK	QUAL	To explore community nurses' experiences in delivering and supporting oral health care for older people living at home.	15 community nurses (12 women, 3 men) from varied specialities (district nursing, mental health, learning disability, acute care). Band levels ranged from 5 to 8. All registered nurses providing care to older adults at home; employed within the UK's National Health Service.	***Demands***: time pressure ***Resources***: self-efficacy/ confidence, standardised practices, role clarity, interprofessional collaboration, formal education
52	Muramatsu et al. (2018) USA	MIX	To investigate slips, trips and falls (STFs) among HC aides, examining their incidence, circumstances, risk factors and potential prevention strategies.	The survey sample included 741 HC ides, mostly women, African American, with an average age of 44.7 years and varying levels of education. Six focus groups (44 participants) included mostly older, more experienced black women.	***Demands***: workload, safety hazards, physical demands, cognitive demands ***Resources***: employer support, supervisor support, personal safety behaviour ***Outcomes***: STFs, injury, stress

Table 1 of supporting information

Characteristics of included studies

Nº	Citation and country	Study design	Aim	Participants	Key codes
53	Nielsen & Jørgensen (2016) Denmark	QUAL	To explore how HCWs experience meaning in their work to deepen understanding of employee engagement.	16 female home health caregivers (aged 24-50) with 1.5-3.3 years of formal training and 2-5 years of experience providing care to elderly or chronically ill clients who needed help with daily tasks at home.	***Demands***: job insecurity, time pressure<br>***Resources***: meaning, client-carer relationship, benefits<br>***Outcomes***: engagement, frustration, quality of care, mental exhaustion
54	Ollé-Espluga et al. (2024) Spain	MIX	To evaluate the impact of a self-managed team model in municipal HBC services on workers' conditions, health, and well-being.	116 HCWs in the quantitative study (79.5% family workers, 20.5% cleaning assistants; 95% women; aged mostly 41–55; 55–61% foreign-born). All employed in the public sector. 10 HCWs (9 women, 1 man) participated in qualitative interviews.	***Demands***: workload and responsibility<br>***Resources***: supervision, self-organising teams, autonomy, co-worker support<br>***Outcomes***: physical fatigue, distress
55	Oomkens et al. (2016) The Netherlands	QUAN	To examine how performance-based contracting, management support and professionalism are connected in HBC.	147 HCWs (nurses and home helps providing personal care services). Majority female.	***Demands***: strict registration rules, cost-efficiency measures<br>***Resources***: autonomy, management support, intrinsic job satisfaction
56	Parella et al. (2024) Spain	QUAL	To investigate how psychosocial working conditions affect the physical and mental health of live-in migrant domestic workers.	22 live-in migrant women domestic workers (interviewed in Madrid and Barcelona); aged 21–70; mainly from Latin America (Bolivia, Honduras, Peru, Ecuador, Paraguay, Colombia, El Salvador); many in irregular legal status; all employed directly by families in live-in arrangements.	***Demands***: employment insecurity, emotional demands, abuse, flexibility<br>***Resources***: client-carer relationship, non-work social support<br>***Outcomes***: health and safety
57	Petersen & Melzer (2026) Germany	QUAN	To explore the associations between the work circumstances of home care nurses and their mental health.	Most of the 972 home care nurses were women (83%), and the mean age was 46. All had completed professional nursing education, and 47% had management tasks.	***Demands***: emotional demands, hiding emotions, time pressure<br>***Resources***: supportive leadership, co-worker support<br>***Outcomes***: mental strain
58	Pinto et al. (2022) Canada	QUAN	To examine the characteristics of the personal support worker (PSW) workforce, their working conditions and job security, and to explore the health of PSWs and how precarious employment affects it.	634 PSWs in a diverse range of settings, primarily in home care (43.9%) and long-term care (34.5%), with others in hospitals or shelters (21.6%). Only HCWs were included in the synthesis; they were predominantly black women, aged 30-49 years, born outside Canada, with a university degree or higher, and from low-income households.	***Demands***: precarious employment, discrimination<br>***Resources***: personal safety, support, sense of belonging in the community<br>***Outcomes***: health condition, life satisfaction, stress

Table 1 of supporting information

Characteristics of included studies

Nº	Citation and country	Study design	Aim	Participants	Key codes
59	Quinn et al. (2016) USA	QUAN	To assess the occupational safety and health (OSH) hazards and benefits of different HBC working conditions, and to compare the OSH experiences of HBCs employed through different medical and social service systems.	1,249 home care aides. Predominantly female, average age 47, born outside the US, and with 2 to 9 years tenure with current employer.	***Demands***: hazardous work environment<br>***Resources***: meaning, control over schedule, autonomy, job security, safety climate<br>***Outcomes***: injury, musculoskeletal well-being
60	Rahnfeld et al. (2016) Germany	QUAN	To determine the association of care setting (nursing home vs. home care) with the turnover intentions of geriatric nurses.	172 home care workers and 106 nursing staff in institutions. Registered nurses and nursing auxiliaries, mostly female, working in HBC for public/non-profit organisations on full-time, fixed-term contracts.	***Demands***: time pressure, social conflicts<br>***Resources***: task identity, social support<br>***Outcomes***: general health, job satisfaction, turnover intentions
61	Ravenswood et al. (2017) Aotearoa (NZ)	QUAN	To examine the prevalence and impact of client-initiated abuse on HBC and community workers, exploring links with job demands, training, job satisfaction and turnover intentions within the JD-R framework.	574 home and community care workers. Mostly female, average age 55.5, with higher education and between 0 and 11-years' experience in the sector.	***Demands***: work intensity, abuse<br>***Resources***: on-the-job training<br>***Outcomes***: job satisfaction, intentions to leave
62	Rong et al. (2022) China	QUAN	To examine factors influencing job satisfaction of HCWs, in Shanghai.	285 migrant HCWs caring for older individuals. Predominantly female, median age 51 years, married and with a secondary school education or less.	***Demands***: lack of employer benefits<br>***Resources***: nature of client-care worker relationship<br>***Outcomes***: job satisfaction, burnout
63	Ruotsalainen et al. (2020) Finland	MIX	To examine how challenges, stressors, teamwork and management affect HCWs' well-being, job satisfaction and perceived quality of care, and to explore their daily work experiences.	121 HCWs, mostly nurses, average age 40.6 years. Most had vocational training. Fifteen staff (all women) took part in the follow-up interviews - 14 practical nurses and one registered nurse.	***Demands***: working alone, interruptions at work, time pressure<br>***Resources***: organisational justice, teamwork, job control, employer support<br>***Outcomes***: job satisfaction, stress, psychological distress, quality of care, physical health
64	Ruotsalainen et al. (2023) Finland	QUAN	To test whether care workers in self-organising teams report higher job satisfaction and lower turnover intentions than those in non-self-organising teams, and if psychosocial factors mediate these effects.	600 care workers (nursing staff, therapists, managers, others). Predominantly female, 43.8 years old and working as home care nurses.	***Demands***: time pressure<br>***Resources***: self-organising teamwork, autonomy, skill discretion<br>***Outcomes***: job satisfaction, turnover intentions, job strain

Table 1 of supporting information

Characteristics of included studies

Nº	Citation and country	Study design	Aim	Participants	Key codes
65	Schoenfisch et al. (2017) USA	MIX	To describe the occupational safety, support resources, and injury experience of unionised HC aides, and to compare between individual providers and agency providers.	43,394 HC aides. Predominantly female, average age 46.1 years, and employed by an individual provider. 18 HC aides took part in the focus groups.	***Demands***: safety hazards, physical demands, tasks beyond duties, workplace violence<br>***Resources***: supervisor support, occupational esteem<br>***Outcomes***: MSDs injury, STFs
66	Simon et al. (2008) EU: BE, DE, FR, IT, NL, PL, and SK	QUAN	To examine how physical and psychosocial risk factors are associated with neck/back pain disability and to assess the influence of the type of healthcare setting (hospital, nursing home, home care) in EU countries.	Workers in hospitals (16,770), nursing homes (2,140) and home care (2,606); only HCWs were included in the review - who were mainly female, aged 30-44, working as registered nurses, auxiliaries or support staff.	***Demands***: physical, lack of technical lifting aids<br>***Resources***: control over working environment<br>***Outcomes***: musculoskeletal, job strain
67	Sims-Gould et al. (2013) Canada	QUAL	To explore the types of crises that home support workers face and how they respond to and manage them.	The 118 home support workers provided personal, emotional and social care, and direct assistance with activities of daily living. Mostly female, 50 years old, university or college educated, 12 years in the profession, seeing 4 patients per day and working full time.	***Demands***: crisis or complex problems, safety hazards, conflict, tasks beyond duties, language barrier, death, uncertainty<br>***Resources***: coping strategies, benefits, flexibility<br>***Outcomes***: fear, stress
68	Sjöberg et al. (2020) Sweden	QUAN	To investigate how perceived workload affects HCWs' health-related quality of life and whether psychosocial factors influence this relationship.	1,029 HCWs (eligible responses), mainly assistant nurses, female, married, with a permanent contract, average age 41 years, and more than 5 years working in the sector.	***Demands***: quantitative demands, learning demands<br>***Resources***: social support, control<br>***Outcomes***: health-related quality of life
69	Solano-Kamaiko et al. (2025) USA	MIX	To investigate the feasibility and utility of using activity tracking devices to provide HCWs with detailed awareness of and insights into the daily activities affecting their health and wellbeing.	17 HCWs, mostly women from racialised backgrounds. They were aged between 29 and 68, had an average of 11.6 years' experience, and provided personal care, domestic tasks, and basic health-related support in the home.	***Demands***: precarious employment, workload pressures, physical demands<br>***Resources***: autonomy, co-worker support<br>***Outcomes***: general health, recovery
70	Stawnychy et al. (2023) USA	QUAN	To find out what affects HCWs' self-efficacy in contributing to their patients' heart failure self-care.	328 HCWs (home health aides, attendants, and personal care aides) caring for adults with heart failure. Predominantly female, non-Hispanic black, with a mean age of 48 years and a mean tenure of 9 years.	***Demands***: clients' diagnosis<br>***Resources:*** previous HF-specific training, self-care self-efficacy and carer preparedness<br>***Outcomes***: job satisfaction

Table 1 of supporting information

Characteristics of included studies

Nº	Citation and country	Study design	Aim	Participants	Key codes
71	Sterling et al. (2018) USA	QUAL	To explore HCWs' perspectives on caring for adults with heart failure, assess their training and role in client self-care, and identify related challenges and needs.	46 HCWs (mean age 49 years), mostly Hispanic women. The majority had at least a high school education, averaged 16 years of experience, and spent approximately 3.5 days per week caring for heart failure patients.	***Demands***: physical demands, overwork, emotional demands, precariousness, job insecurity, death, isolation<br>***Resources***: occupational esteem, employer support, appreciation, supervisor support, meaning<br>***Outcomes***: mental exhaustion, job satisfaction, fear, worry, frustration
72	Strandell (2020) Sweden	QUAN	To compare the work content, conditions and challenges of Swedish HCWs in 2005 and 2015, and to examine links with sector changes such as deinstitutionalisation, cost pressures and New Public Management reforms.	371 HCWs, mainly women (although the proportion of men increased between 2005 and 2015), middle-aged, mostly born outside Sweden, with more than 10 years' experience and formal care training. Most worked full-time (35+ hours/week) in the public sector.	***Demands***: workload, time pressure<br>***Resources***: Autonomy, social support, rewarding client-care worker relationship, self-adequacy<br>***Outcomes***: physical and mental exhaustion
73	Swedberg et al. (2013) Sweden	QUAL	To explore how HC assistants, as paraprofessionals with limited training, met the challenges of caring for high-need patients at home without hospital support.	19 HC assistants (mostly female, average age 36.3) employed in 24-hour care agencies. All were municipal employees with varying levels of education, ranging from a 3-year upper secondary health and social care programme to shorter courses or employer-provided training.	***Demands***: working environment, disrespect, work alone, safety hazards, complexity<br>***Resources***: training, skill, supervision, anticipation, coordination, balancing client-carer relationship, social support<br>***Outcomes***: stress, work-life spillover
74	Tourangeau et al. (2014) Canada	QUAL	To identify factors that influence Canadian home care nurses' intention to remain in the profession.	50 home care nurses. Mostly female, from the baby boomer generation and working for a not-for-profit organisation.	***Demands***: workload, time pressure, overwork, use of technology, job insecurity, precariousness<br>***Resources***: variety, autonomy, social support, benefits, control, meaning, unionisation<br>***Outcomes***: intention to remain employed, quality of care, frustration, stress, work-life spillover
75	Van de Weerdt & Baratta (2015) France	QUAL	The study analyses the working conditions of HCWs and their impact on job satisfaction, well-being, emotions, relationships and occupational stress.	8 nurses, 7 nursing assistants, 3 coordinating nurses and 3 secretaries.	***Demands***: time pressure, difficult clients, emotional demands, safety hazards<br>***Resources***: emotional regulation, social support, meaning, benefits<br>***Outcomes***: job satisfaction, emotional well-being, stress, quality of care

Table 1 of supporting information

Characteristics of included studies

Nº	Citation and country	Study design	Aim	Participants	Key codes
76	Van Waeyenberg et al. (2015) Belgium	QUAN	To explore how the quality and frequency of supervisory feedback, as well as home nurses' own self-efficacy, affect their intention to leave their job.	312 home nurses from a non-profit home care organisation, 99% were women, aged between 21 and 63 years old and had an average organisational tenure of 12.6 years. Most worked part-time (77%).	***Resources***: supportive leadership, self-efficacy ***Outcomes***: turnover intentions
77	Wills et al. (2016) USA	QUAL	To describe how HCWs identify and manage health and safety hazards in clients' homes, focusing on the dilemmas they face, their decision-making processes and the quality of those decisions.	68 Home care workers. The majority were female, fluent in spoken and written English, aged between 22 and 73 years and had between 1 and 36 years of home care experience.	***Demands***: safety hazards, physical demands, workload ***Resources***: access to lifting equipment, personal safety behaviour, training ***Outcomes***: physical ill-being
78	Zhang et al. (2022) China	QUAL	To investigate the challenges faced by HCWs providing end-of-life care at home.	13 home care nurses, aged 25 to 53 (mean 37.2). Their previous nursing experience ranged from 3 to 29 years, with 3 to 12 months in HBC. 3 nurses were tenured (life-long contract), while 10 were non-tenured.	***Demands***: illegitimate tasks ***Resources***: skill, benefits, development, self-efficacy, meaning ***Outcomes***: motivation, quality of care, turnover intentions
79	Zoeckler (2018) USA	QUAL	To explore occupational stressors for paid home care workers, focusing on agency characteristics and workers' personal experiences.	25 HCWs providing routine healthcare and basic monitoring of the patient's health status. All but one were women. The majority were white, US-born, married, and 48 years old on average.	***Demands***: discrimination, death, schedule demands, difficult clients, precariousness, emotional demands, violence, time pressure ***Resources***: occupational esteem, benefits, training, social support, meaning, HR practices, autonomy ***Outcomes***: injury, stress, mental exhaustion, turnover, quality of care, job satisfaction

Note: QUAN – quantitative, MIX – mixed-methods, QUAL – qualitative; HCWs – Home Care Workers

Supplementary material – File 4: MMAT tool 2018 version for selected studies

MMAT (quality appraisal for quantitative non-randomized studies)

Study	Citation	S1	S2	3.1.	3.2.	3.3.	3.4.	3.5
3	(Aronsson et al., 2014)	Y	Y	C	Y	Y	Y	Y
5	(Ayalon, 2012)	Y	Y	N	Y	Y	Y	Y
7	(Bakker et al., 2003)	Y	Y	Y	Y	Y	Y	Y
14	(Chang et al., 2025)	Y	Y	N	Y	Y	N	Y
16	(Cindrić & Malnar, 2025)	Y	Y	Y	Y	Y	N	C
20	(Fernández-Carrasco et al., 2022)	Y	Y	N	Y	Y	N	Y
24	(Green & Ayalon, 2018)	Y	Y	Y	Y	Y	N	Y
26	(Hanson et al., 2015)	Y	Y	C	Y	Y	Y	Y
36	(Larsson et al., 2012)	Y	Y	C	Y	Y	Y	Y
38	(Lee & Jang, 2016)	Y	Y	N	Y	Y	Y	Y
39	(Lee & Oh, 2023)	Y	Y	C	Y	Y	Y	Y
41	(Lindholm et al., 2021)	Y	Y	C	Y	Y	Y	Y
55	(Oomkens et al., 2016)	Y	Y	C	Y	Y	Y	Y
57	(Petersen & Melzer, 2026)	Y	Y	N	Y	Y	N	N
58	(Pinto et al., 2022)	Y	Y	C	Y	Y	Y	Y
60	(Rahnfeld et al., 2016)	Y	Y	N	Y	Y	Y	Y
61	(Ravenswood et al., 2017)	Y	Y	N	Y	Y	Y	Y
62	(Rong et al., 2022)	Y	Y	C	Y	Y	Y	Y
64	(Ruotsalainen et al., 2023)	Y	Y	C	Y	Y	Y	Y
66	(Simon et al., 2008)	Y	Y	Y	Y	Y	Y	Y
68	(Sjöberg et al., 2020)	Y	Y	C	Y	Y	Y	Y
70	(Stawnychy et al., 2023)	Y	Y	C	Y	Y	Y	Y
72	(Strandell, 2020)	Y	Y	Y	Y	Y	Y	Y
76	(Van Waeyenberg et al., 2015)	Y	Y	N	Y	Y	N	Y

Note: S1. Are there clear research questions? S2. Do the collected data allow to address the research questions? 3.1. Are the participants representative of the target population? 3.2. Are measurements appropriate regarding both the outcome and intervention (or exposure)? 3.3. Are there complete outcome data? 3.4.

Are the confounders accounted for in the design and analysis? 3.5. During the study period, is the intervention administered (or exposure occurred) as intended? Y = Yes, N = No, C = Can't tell

MMAT (quality appraisal for quantitative descriptive studies)

Study	Citation	S1	S2	4.1.	4.2.	4.3.	4.4.	4.5
1	(Agbonifo et al., 2017)	Y	Y	N	N	C	C	Y
6	(Bagnasco et al., 2024)	Y	Y	C	N	Y	C	Y
23	(Ghoroubi et al., 2023)	Y	Y	Y	Y	Y	Y	Y
27	(Hittle et al., 2016)	Y	Y	N	N	C	C	Y
28	(Hsu & Chen, 2025)	Y	Y	C	N	Y	N	Y
37	(Larsson et al., 2013)	Y	Y	Y	C	Y	N	Y
43	(Lohne et al., 2024)	Y	Y	C	N	Y	N	Y
59	(Quinn et al., 2016)	Y	Y	Y	C	Y	C	Y

Note: S1. Are there clear research questions? S2. Do the collected data allow to address the research questions? 4.1. Is the sampling strategy relevant to address the research question? 4.2. Is the sample representative of the target population? 4.3. Are the measurements appropriate? 4.4. Is the risk of nonresponse bias low? 4.5. Is the statistical analysis appropriate to answer the research question? Y = Yes, N = No, C = Can't tell

MMAT (quality appraisal for mixed-methods studies)

Study	Citation	S1	S2	5.1.	5.2.	5.3.	5.4.	5.5
2	(Andersen & Westgaard, 2015)	Y	Y	Y	Y	Y	Y	Y
4	(Ayalon, 2009)	Y	Y	Y	Y	Y	Y	N
10	(Butler, 2018)	Y	Y	Y	Y	Y	Y	Y
11	(Butler et al., 2010)	Y	Y	Y	Y	Y	Y	Y
12	(Butler et al., 2012)	Y	Y	Y	Y	Y	Y	Y
15	(Chowdhury & Gutman, 2012)	Y	Y	Y	Y	Y	Y	N
17	(Delp & Muntaner, 2011)	Y	Y	Y	Y	Y	Y	C
18	(Denton et al., 2002)	Y	Y	Y	Y	Y	Y	Y
21	(Fleming & Taylor, 2006)	Y	Y	Y	Y	Y	Y	Y
31	(Karlsson et al., 2020)	Y	Y	Y	Y	Y	Y	Y
50	(Minguela-Recover et al., 2022)	Y	Y	Y	Y	Y	Y	Y
52	(Muramatsu et al., 2018)	Y	Y	Y	Y	Y	Y	Y
54	(Ollé-Espluga et al., 2024)	Y	Y	Y	Y	Y	Y	Y
63	(Ruotsalainen et al., 2020)	Y	Y	Y	Y	Y	Y	Y
65	(Schoenfisch et al., 2017)	Y	Y	Y	Y	Y	Y	Y
69	(Solano-Kamaiko et al., 2025)	Y	Y	Y	Y	Y	Y	Y

Note: S1. Are there clear research questions? S2. Do the collected data allow to address the research questions? 5.1. Is there an adequate rationale for using a mixed methods design to address the research question? 5.2. Are the different components of the study effectively integrated to answer the research question? 5.3. Are the outputs of the integration of qualitative and quantitative components adequately interpreted? 5.4. Are divergences and inconsistencies between quantitative and qualitative results adequately addressed? 5.5. Do the different components of the study adhere to the quality criteria of each tradition of the methods involved? Y = Yes, N = No, C = Can't tell

MMAT (quality appraisal for qualitative studies)

Study	Citation	S1	S2	1.1.	1.2.	1.3.	1.4.	1.5
8	(Barken et al., 2015)	Y	Y	Y	Y	Y	Y	Y
9	(Brulin et al., 2000)	Y	Y	Y	Y	Y	Y	Y
13	(Cacciapuoti et al., 2025)	Y	Y	Y	Y	Y	Y	Y
19	(Denton et al., 2015)	Y	Y	Y	Y	Y	Y	Y
22	(Franzosa et al., 2019)	Y	Y	Y	Y	Y	Y	Y
25	(Gusoff et al., 2025)	Y	Y	Y	Y	Y	Y	Y
29	(Janssen & Abbott, 2023)	Y	Y	Y	Y	Y	Y	Y
30	(Jepsen et al., 2025)	Y	Y	Y	Y	Y	Y	Y
32	(Kelly et al., 2024)	Y	Y	Y	Y	Y	Y	Y
33	(Koivula et al., 2016)	Y	Y	Y	Y	Y	Y	Y
34	(Kriegsmann-Rabe et al., 2023)	Y	Y	Y	Y	Y	Y	Y
35	(Kusmaul et al., 2020)	Y	Y	Y	Y	Y	Y	Y
40	(Liaset et al., 2024)	Y	Y	Y	Y	Y	Y	Y
42	(Lindquist et al., 2012)	Y	Y	Y	Y	Y	Y	Y
44	(Love et al., 2017)	Y	Y	Y	Y	Y	Y	Y
45	(Markkanen et al., 2007)	Y	Y	Y	Y	N	Y	Y
46	(Markkanen et al., 2014)	Y	Y	Y	Y	Y	Y	Y
47	(Markkanen et al., 2017)	Y	Y	Y	Y	Y	Y	Y
48	(Martínez-Buján & Moré, 2024)	Y	Y	Y	Y	Y	Y	Y
49	(Martínez-de la Torre et al., 2025)	Y	Y	Y	Y	Y	Y	Y
51	(Mitchell et al., 2023)	Y	Y	Y	Y	Y	Y	Y
53	(Nielsen & Jørgensen, 2016)	Y	Y	Y	Y	Y	Y	Y
56	(Parella et al., 2024)	Y	Y	Y	Y	Y	Y	Y
67	(Sims-Gould et al., 2013)	Y	Y	Y	Y	Y	Y	Y
71	(Sterling et al., 2018)	Y	Y	Y	Y	Y	Y	Y
73	(Swedberg et al., 2013)	Y	Y	Y	Y	Y	Y	Y
74	(Tourangeau et al., 2014)	Y	Y	Y	Y	Y	Y	Y
75	(Van de Weerdt & Baratta, 2015)	Y	Y	Y	Y	Y	Y	Y
77	(Wills et al., 2016)	Y	Y	Y	Y	Y	Y	Y
78	(Zhang et al., 2022)	Y	Y	Y	Y	Y	Y	Y

79	(Zoeckler, 2018)	Y	Y	Y	Y	Y	Y	Y

Note: S1. Are there clear research questions? S2. Do the collected data allow to address the research questions? 1.1. Is the qualitative approach appropriate to answer the research question? 1.2. Are the qualitative data collection methods adequate to address the research question? 1.3. Are the findings adequately derived from the data? 1.4. Is the interpretation of results sufficiently substantiated by data? 1.5. Is there coherence between qualitative data sources, collection, analysis, and interpretatio
